# Genome Wide Expression Analysis Suggests Perturbation of Vascular Homeostasis during High Altitude Pulmonary Edema

**DOI:** 10.1371/journal.pone.0085902

**Published:** 2014-01-22

**Authors:** Manish Sharma, Shashi Bala Singh, Soma Sarkar

**Affiliations:** Molecular Biology Division, Defence Institute of Physiology And Allied Sciences (DIPAS), DRDO, Delhi, India; Vanderbilt University Medical Center, United States of America

## Abstract

**Background:**

High altitude pulmonary edema (HAPE) is a life-threatening form of non-cardiogenic edema which occurs in unacclimatized but otherwise normal individuals within two to four days after rapid ascent to altitude beyond 3000 m. The precise pathoetiology and inciting mechanisms regulating HAPE remain unclear.

**Methodology/Principle findings:**

We performed global gene expression profiling in individuals with established HAPE compared to acclimatized individuals. Our data suggests concurrent modulation of multiple pathways which regulate vascular homeostasis and consequently lung fluid dynamics. These pathways included those which regulate vasoconstriction through smooth muscle contraction, cellular actin cytoskeleton rearrangements and endothelial permeability/dysfunction. Some notable genes within these pathways included *MYLK*; rho family members *ARGEF11*, *ARHGAP24*; cell adhesion molecules such as *CLDN6*, *CLDN23*, *PXN* and *VCAM1* besides other signaling intermediates. Further, several important regulators of systemic/pulmonary hypertension including *ADRA1D, ECE1,* and *EDNRA* were upregulated in HAPE. We also observed significant upregulation of genes involved in paracrine signaling through chemokines and lymphocyte activation pathways during HAPE represented by transcripts of *TNF*, *JAK2, MAP2K2, MAP2K7, MAPK10*, *PLCB1*, *ARAF*, *SOS1*, *PAK3* and *RELA* amongst others. Perturbation of such pathways can potentially skew vascular homeostatic equilibrium towards altered vascular permeability. Additionally, differential regulation of hypoxia-sensing, hypoxia-response and OXPHOS pathway genes in individuals with HAPE were also observed.

**Conclusions/Significance:**

Our data reveals specific components of the complex molecular circuitry underlying HAPE. We show concurrent perturbation of multiple pathways regulating vascular homeostasis and suggest multi-genic nature of regulation of HAPE.

## Introduction

High altitude pulmonary edema (HAPE) is a life threatening clinical condition that occurs in otherwise healthy individuals who rapidly ascend to high altitude (above 3000 m). It is the major cause of death related to high altitude exposure. Though notable physiological and clinical studies in past two decades have underscored the importance of ‘acclimatization schedules’ in preventing HAPE, the individual susceptibility beyond acclimatization remains far from being deciphered. A prior history of HAPE [1], rapid ascent to high altitude [2], strenuous exercise at high altitude [3], preexisting respiratory infection [4] and genetic factors [5] are some of the proposed reasons for HAPE. Previous studies have shown exaggerated, unsustainable and non-uniform pulmonary vasoconstriction, leading to mechanically induced breaks at blood-gas barrier, to be concomittant with HAPE [6]. Elevated capillary pressure possibly due to inhomogeneous hypoxic pulmonary vasoconstriction (HPV) [7], [8], [9], transarteriolar leakage [10] and/or hypoxic venoconstriction [11] in pulmonary circulation are known to be causally linked to HAPE. Augmented hypoxic pulmonary vasoconstrictor (HPV) response to hypoxia [12], [13] and increased pulmonary artery systolic pressure (PASP) during exercise in normoxia [14] have also been reported in susceptible individuals. Reduced pulmonary synthesis of endothelium-derived vasodilator nitric oxide (NO) and impairment of NO mediated pulmonary vasodilation [15], [16] are some of the other factors of significance for HAPE susceptibility.

Amongst the aforementioned factors, it is difficult to identify primary and secondary factors which regulate individual HAPE susceptibility. Furthermore, the involvement of inflammation in primary etiology of HAPE remains unclear, suggesting complexity of possible underlying mechanisms. It is likely that increased hydrostatic pressure, inflammatory response or a combination of both could be important [6]. Notably, individuals who develop HAPE run a significant risk of recurrence suggesting involvement of genetic component in its etiology although little is known about the genetic basis of HAPE. An understanding of basic molecular mechanism underlying HAPE is thus necessary not only for designing of appropriate preventive or prophylactic strategies but more importantly for evaluating susceptibility. In the present study, we utilized a genome-wide approach to identify differentially expressed genes and pathways from peripheral blood of individuals who developed HAPE after acute induction to high altitude. Peripheral blood cells have been suggested to serve as a representative sample of the systemic state allowing for evaluation and profiling of multiple pathological and physiological pathways [17]. We implicate multiple genes within different pathways composing a complex network regulating vascular homeostasis and possibly, in consequence, lung fluid dynamics during HAPE.

## Experimental Procedures

### Study Design

The study population consisted of male sea level residents of Indian origin (n = 17) who had developed HAPE after air induction (ascent by flying) to high altitude (Leh situated at 3250 m). Four volunteers were fresh inductees while the rest were re-inductees who travelled to high altitude after spending their leave period at sea level (Supplementary Table S1). After arrival at the Leh altitude, they were undergoing acclimatization as per prescribed acclimatization schedule but developed HAPE within 24–96 hrs. One individual developed HAPE after 120 hours. They were immediately admitted to Leh hospital and provided medical aid and oxygen supplementation. Diagnosis of HAPE was done by the specialist medical officers at the hospital who attended to the patients based on presence of pulmonary rales, cyanosis and chest radiography. The clinical symptoms included cough, dyspnoea at rest, breathlessness, chest tightening and congestion. The control group consisted of age, sex and duration matched lowlanders (n = 14) who were also inducted under similar conditions, underwent similar acclimatization schedule and did not develop HAPE (these volunteers were considered acclimatized). All the volunteers were healthy at sea level as per their annual medical record and did not suffer from any congenital anomaly or cardiovascular disease. The study was approved by ‘Institute Ethics Committee of Defence Institute of Physiology and Allied Sciences (DIPAS), Delhi’ and informed written consent was obtained from all volunteers. Clinical parameters of heart rate, systolic and diastolic blood pressure and arterial oxygen saturation were measured in HAPE individuals before oxygen supplementation and from acclimatized individuals before drawing blood.

### RNA isolation and Aminoallyl labeling

Blood samples from HAPE individuals were collected immediately on admission prior to medication and oxygen supplementation except for three individuals from whom samples were collected 8 hrs after admission to hospital and stabilization of patients’ condition (Supplementary Table S1). Blood samples were collected from acclimatized individuals within 45–72 hrs of arrival to high altitude except one (Supplementary Table S1). About 7 ml of venous whole blood was collected in PAXgene Blood RNA Tubes (PAXgene, PreAnalytiX, Hombrehtikon, Switzerland, distributed by Qiagen, catalog no. 762125 ex US) at room temperature using a blood collection accessory (Beckton Dickinson) and stored at 4°C. Within two days of collection, samples were transported on ice to the laboratory in Delhi situated at sea level. Isolation of total cellular RNA was done utilizing PAXgene Blood RNA kit (PreAnalytiX) as per manufacturer’s protocol. An on-column DNase digestion was carried out for the removal of DNA using RNase-free DNase set (Qiagen) and purified RNA stored at –80°C. RNA concentration and quality was evaluated by measuring absorbance at 260 and 280 nm using a NanoDrop (NanoDrop, USA) and electrophoretic analysis on Agilent 2100 Bioanalyzer (Agilent Technologies Inc., Palo Alto, CA ) prior to labeling. Probes were generated by aminoallyl labeling using Amino AllylMessageAmp™ II aRNA Amplification Kit (Ambion, Life Technologies Corporation) and Cy3™ post labeling reactive dye pack (GE Healthcare, UK).

### Microarray hybridization and image analysis

**Figure 1:**
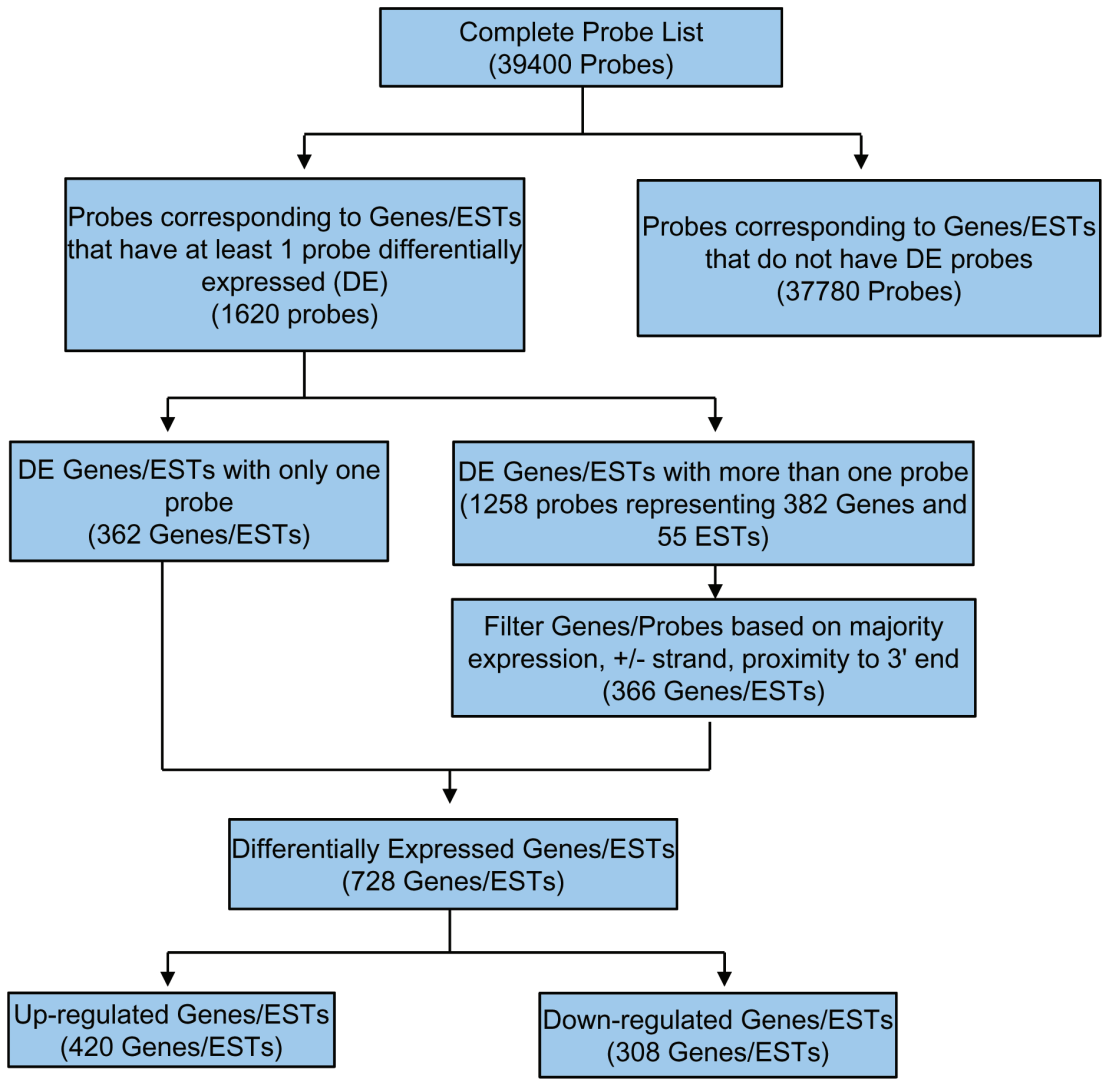
Work flow of gene expression analysis. The figure represents scheme of extraction of differentially expressed genes (up regulated: 420, down regulated: 308) from the raw microarray data.

Hybridization to Human Whole Genome oligochip 40K (Ocimum, Hyderabad, India) was carried out as per manufacturer’s protocol (Ocimum, Hyderabad, India) and data submitted to GEO (Accession number: GSE52209). Experimental design, sampling, hybridization and data analysis were performed in compliance with Minimum Information About a Microarray Experiment (MIAME) guidelines (Supplementary Table S2). Each array representing a sample was scanned for three different PMT settings (40, 50 and 60) on Affymetrix 428™ Scanner. The images were loaded into the image analysis software (Imagene) and the transcript levels quantified. Initially, the data consisted of 40,320 probes, including empty spots and control probes which were removed leaving behind 39,400 probes. Out of these, 1620 probes corresponding to genes/ESTs that had at least one probe differentially expressed were chosen for further downstream filtering (Figure 1). Probes were further filtered based on majority expression, +/– strand and proximity to 3’ end. The missing values were imputed by K-nearest neighbor (KNN) method. The intensity data for each sample was transformed on log_2_ scale. Quantile normalization was used to overcome the systematic variation across the arrays. Three HAPE samples showed slightly different distribution profiles (bimodal) compared to other samples, however, for normalization, all samples were retained and distribution profiles obtained.

### Differential expression analysis

Linear modeling approach was used for assessment of differential expression. The LIMMA [18] library from R-Bioconductor package was utilized to construct the linear models and gene wise linear models were set based on the experimental design. Analysis involved estimating consistent, closed form estimators for the hyper parameters using the marginal distributions of selected statistics. ‘Robust linear method’ (rlm), which is robust to outliers, measurement errors and other data irregularities, was used for estimating the model coefficients. These estimators were based on the two-step weighted least squares method, where weights were adaptively computed using the empirical distribution of residuals obtained from initial robust fit. To obtain differentially expressed genes, moderated t-statistic was used involving posterior residual standard deviations rather than the ordinary standard deviations. The design matrix consisted of two conditions/groups. The difference in the expression level of samples in the two conditions was studied by setting appropriate contrasts. The multiplicity of testing was performed using Benjamin & Hochberg (BH) correction adjusting for the false discovery rate (FDR) since each analysis involved large number of tests. The threshold adjusted p-value was set to 0.05 and the fold change threshold was set to 1.5. These settings were retained throughout the analysis to select gene list across the comparisons. Expression levels of HAPE samples were compared with those of acclimatized control samples. The up/down regulation of a gene was decided only if the difference in the expression was statistically significant (p≤0.05) and the absolute fold-change was greater than 1.5. If either of the criterions were not fulfilled, then the gene expression was treated as insignificant. Afold change of 1.5 was utilized in order to retain maximum biological information present in the data set. The data on log fold change and the p-value was further used to generate volcano plot (Supplementary Figure S1). A two-way hierarchical clustering was performed using the up and down regulated genes for the selected probes. Euclidean distance measure and average linkage method were used to obtain the relatedness of samples and genes and generation of heat maps.

### Gene Enrichment analysis and Functional Annotation Clustering

The differentially expressed genes were studied for their overabundance in different GO terms as well as Pathways. The GO terms were categorized into biological process, molecular function and cellular component. The overabundance of a particular term was decided based on the number of significant genes in the analysis, the number of significant genes relevant to the term, the total number of genes for the organism and the number of genes that were relevant to the term for the organism. Fisher’s exact test was used to determine the significance of each GO term; the significance of a term (p≤0.05) was an indicator that it was enriched with genes. Functional Annotation Clustering was achieved using the Database for Annotation, Visualization and Integrated Discovery (DAVID) [19]. All genes which were identified to be both significantly (p≤0.05) and numerically (±1.5 fold change) expressed and also assigned with an official gene symbol and accession number, were used in the analysis by DAVID. Intergenic network (p≤0.05) was extracted using Pathway Miner [20] for understanding the functional interactions between the differentially expressed genes.

### Real-time quantitative validation of microarray data with TaqMan Low Density Array (TLDA)

Confirmation of microarray results was performed on randomly selected differentially expressed genes (n = 20, FC>1.5, p≤0.05) by Real Time Polymerase Chain Reaction on a microfluidic card assay system (TLDA) of Applied Biosystems (Foster, CA) using the same samples used in microarray experiments. The card included TaqMan probes and primer sets for amplification of target genes and an endogenous control of ribosomal RNA 18S. For each sample, cDNA was prepared with High Capacity Reverse transcription kit (Applied Biosystems). qRT-PCR was performed in duplicate on cDNA samples (200 ng) in each port of TLDA card through 50°C for 2 min, 94.5°C for 10 min, 40 cycles of 97°C for 30 s and 59.7°C for 1 min on ABI PRISM 7900 HT Sequence Detection System (Applied Biosystems, USA). The data was analyzed using comparative cycle threshold method (C_T_ method) 2^−ΔΔCt^ using the RQ software [21].

## Results

The clinical parameters of heart rate (HR), systolic blood pressure (SBP), diastolic blood pressure (DBP) and arterial oxygen saturation (SaO_2_) from HAPE and acclimatized individuals is presented in Supplementary Table S1 and were in the similar range as reported earlier by us [22].

### Global analysis of differentially regulated genes and enriched functional clusters in individuals with HAPE

We utilized RNA extracted from HAPE and acclimatized individuals to perform microarray experiments as detailed in previous section. From these experiments, we found significantly altered expression levels of 728 genes/ESTs (by more than/equal to 1.5 fold change) in peripheral blood cells of individuals with HAPE (Figure 1). Amongst these, 420 genes/ESTs were upregulated and 308 genes/ESTs were downregulated. The complete list of genes along with information for respective biological process, molecular function, cellular localization is shown in Supplementary Table S3.

**Figure 2:**
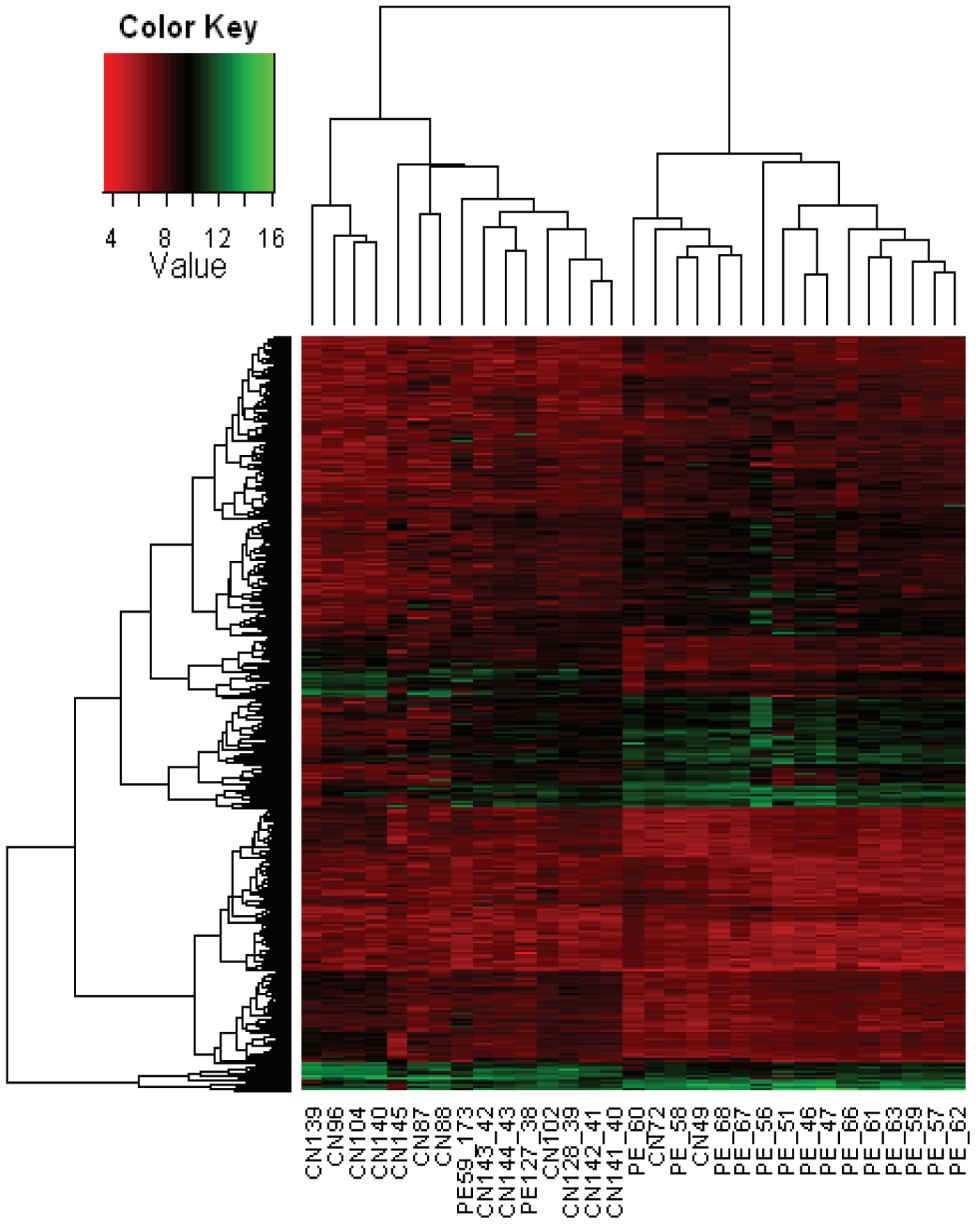
Hierarchical clustering of gene expression data obtained from acclimatized controls (CN) and HAPE (PE) individuals. Hierarchical clustering distinctly separated the two groups of individuals (CN and PE) indicating unique gene expression signatures. Expression values of specific genes are represented by color intensities shown in the reference color key.

**Figure 3:**
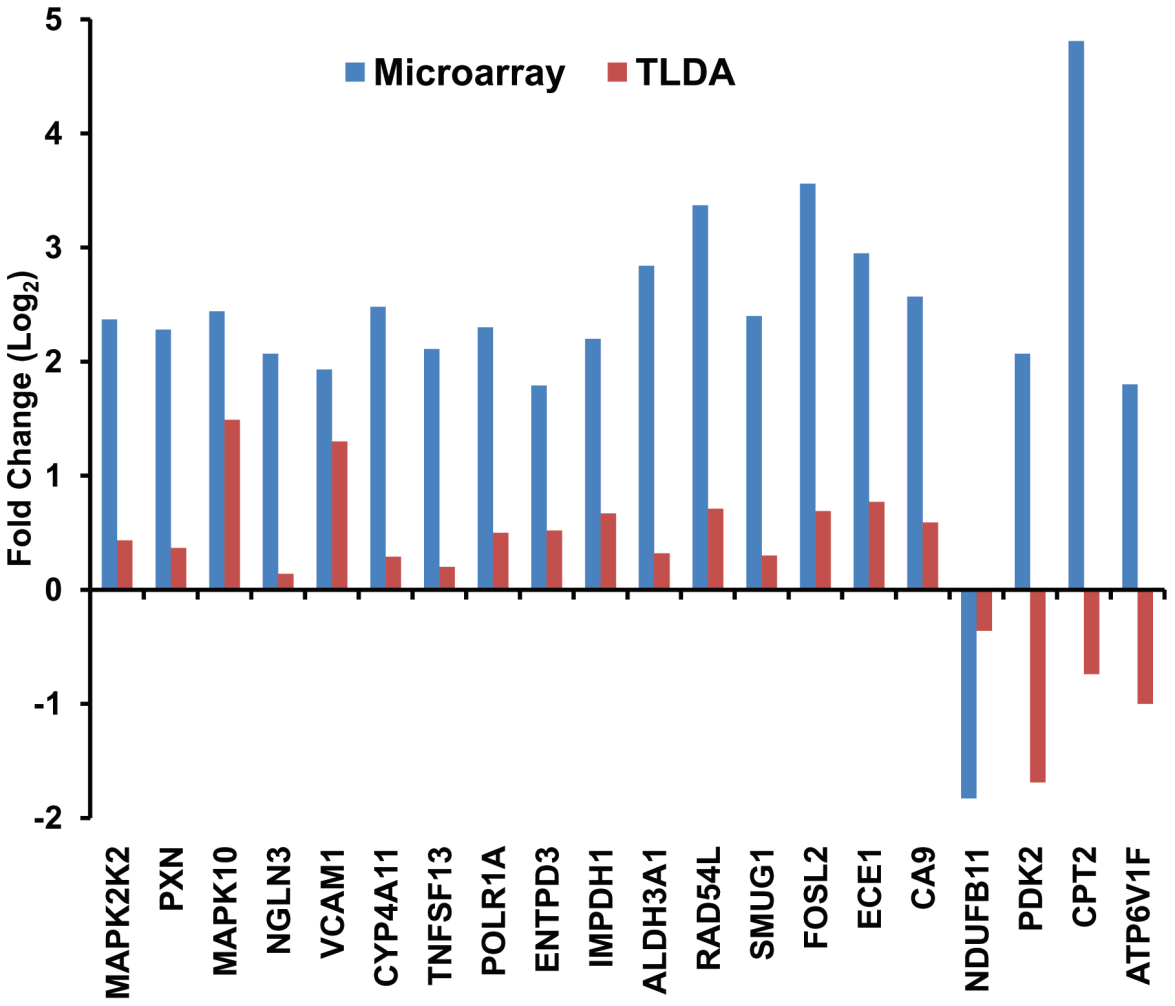
Comparison of relative expression (log2 values) of selected genes obtained by microarray and real time PCR experiments (TLDA). 17 out of 20 genes show similarity of trend on both the platforms.

The hierarchical clustering of this data set distinctly separated the acclimatized lowlanders from individuals with HAPE (Figure 2). We infer these results to be indicative of unique gene expression signatures of two groups of individuals (HAPE and acclimatized) and likely ‘unifying’ molecular responses within specific groups in response to high altitude exposure. Out of the 20 genes selected for validation, a subset of 17 genes yielded positive correlation and showed similarity of trends across the microarray and the RT-PCR platforms attesting overall authenticity of microarray data; only 3 transcripts showed a mismatch of expression between the two detection methods (Figure 3).

**Figure 4:**
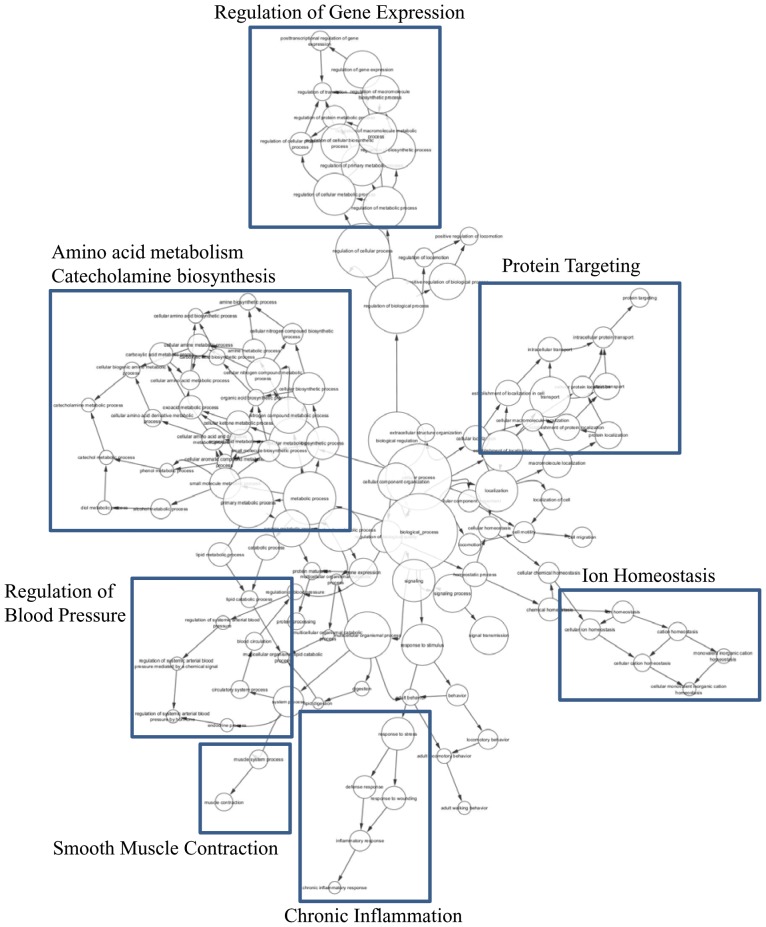
Clustering of GO terms related to biological processes. GO terms were clustered utilizing software BINGO and individual clusters indicated as shown in the figure.

Categorization of the differentially expressed genes based on their biological process, molecular functions and cellular component are shown in Supplementary Figure S2. Maximum number of differentially expressed transcripts in the molecular function category were for binding activity, followed by transcripts involved in catalytic activity. The other differentially expressed transcript categories of molecular function were representative of enzyme regulator activity, transcription regulator activity, molecular transducer activity and transporter activity. We further clustered the GO terms utilizing software ‘BINGO’ and as shown in Figure 4, observed enrichment of seven biological processes. These included amino acid metabolism and catecholamine synthesis, regulation of blood pressure, smooth muscle contraction, chronic inflammation, ion homeostasis, protein targeting and regulation of gene expression. The enrichment of inter-linked biological processes especially, the regulation of catecholamine biosynthesis, blood pressure, smooth muscle contraction and chronic inflammation evoked an interesting possibility suggesting modulation of vascular homeostasis during HAPE.

Further analysis through DAVID revealed a number of biological processes in HAPE individuals which were significantly enriched (p≤0.5, Fold Enrichment >1.1). Some of the critical processes were those involved with oxidative phosphorylation (OXPHOS) and ATP synthesis, ion homeostasis, apoptosis, voltage-gated channel activity, iron-sulfur cluster binding, intra cellular protein transport,TNF-associated molecules, regulation of systemic arterial blood pressure, Ras-guanyl exchange factor activity amongst others. Transcripts associated with smooth muscle contraction, lipid catabolic process, calcium ion transport, actin binding and inflammatory response were also differentially expressed in HAPE individuals. The high stringency functional annotaion cluster is shown in Supplementary Table S4.

### Biological Pathways overrepresented in HAPE

The differentially expressed genes in HAPE were studied for their overabundance in different pathways and are presented in Table 1. We considered p≤0.5 in the DAVID analysis for pathway specific information. Notable pathways found to be affected in HAPE were oxidative phosphorylation (KEGG_pathway hsa:00190), vascular smooth muscle contraction (KEGG_pathway hsa:04270), fatty acid metabolism (KEGG_pathway hsa:00071), VEGF signalling pathway (KEGG_pathway hsa:04370), leucocyte transendothelial migration (has:04670), chemokine signalling pathway (has:04062), Adipocytokine signaling pathway (KEGG_pathway hsa:04920), T cell receptor signaling pathway (KEGG_pathway hsa:04660), gap junction (KEGG_pathway hsa:04540) and cell adhesion molecules (KEGG_pathway hsa:04514). The significance of these pathways in HAPE is discussed in the subsequent sections.

**Table 1:** KEGG Pathway-specific gene transcripts in HAPE data set (Extracted from DAVID Bioinformatics Resource).

KEGG Pathway	P	Accession	Gene Symbol	Gene Name	Fold Change
**hsa00190:Oxidative phosphorylation**	3.82E-04	NM_004231	ATP6V1F	ATPase, H+ transporting, lysosomal 14 kDa, V1 subunit F	1.78
		AC114750	NDUFA10	NADH dehydrogenase (ubiquinone) 1 alpha subcomplex, 10, 42 kDa	1.62
		NM_005006	NDUFS1	NADH dehydrogenase (ubiquinone) Fe-S protein 1, 75 kDa (NADH-coenzyme Q reductase)	1.57
		NM_007103	NDUFV1	NADH dehydrogenase (ubiquinone) flavoprotein 1, 51 kDa	–1.61
		AF363578	ATP6V1C1	ATPase, H+ transporting, lysosomal 42 kDa, V1 subunit C1	–1.64
		NM_006003	UQCRFS1,UQCRFSL1	ubiquinol-cytochrome c reductase, Rieske iron-sulfur polypeptide-like 1; ubiquinol-cytochrome c reductase, Rieske iron-sulfur polypeptide 1	–1.78
		AC002400	NDUFAB1	NADH dehydrogenase (ubiquinone) 1, alpha/beta subcomplex, 1, 8 kDa	–1.8762
		DQ246833	COX1,	Cytochrome c oxidase subunit 1	–2.0502
		DQ246833	COX2,	Cytochrome c oxidase subunit 2	–2.0502
		DQ246833	ATP6, ATP8, COX3,	Cytochrome c oxidase subunit 3; ATP synthase subunit a; ATP synthase protein 8	–2.0502
		DQ246833	ND1,	NADH-ubiquinone oxidoreductase chain 1	–2.0502
		DQ246833	ND2,	NADH-ubiquinone oxidoreductase chain 2	–2.0502
		DQ246833	ND4,ND4L,	NADH-ubiquinone oxidoreductase chain 4L; NADH-ubiquinone oxidoreductase chain 4	–2.0502
		DQ246833	ND5,	NADH-ubiquinone oxidoreductase chain 5	–2.0502
		DQ246833	ND6,	NADH-ubiquinone oxidoreductase chain 6	–2.0502
					
**hsa05012:Parkinson's disease**	3.30E-03	AC114750	NDUFA10,	NADH dehydrogenase (ubiquinone) 1 alpha subcomplex, 10, 42 kDa	1.6258
		NM_005006	NDUFS1,	NADH dehydrogenase (ubiquinone) Fe-S protein 1, 75 kDa (NADH-coenzyme Q reductase)	1.57
		NM_007103	NDUFV1,	NADH dehydrogenase (ubiquinone) flavoprotein 1, 51 kDa	–1.61
		NM_006003	UQCRFS1, UQCRFSL1,	ubiquinol-cytochrome c reductase, Rieske iron-sulfur polypeptide-like 1; ubiquinol-cytochrome c reductase, Rieske iron-sulfur polypeptide 1	–1.78
		AC002400	NDUFAB1,	NADH dehydrogenase (ubiquinone) 1, alpha/beta subcomplex, 1, 8 kDa	–1.8762
		DQ246833	COX1,	Cytochrome c oxidase subunit 1	–2.0502
		DQ246833	COX2,	Cytochrome c oxidase subunit 2	–2.0502
		DQ246833	ATP6, ATP8, COX3,	Cytochrome c oxidase subunit 3; ATP synthase subunit a; ATP synthase protein 8	–2.0502
		DQ246833	ND1,	NADH-ubiquinone oxidoreductase chain 1	–2.0502
		DQ246833	ND2,	NADH-ubiquinone oxidoreductase chain 2	–2.0502
		DQ246833	ND4,ND4L,	NADH-ubiquinone oxidoreductase chain 4L; NADH-ubiquinone oxidoreductase chain 4	–2.0502
		DQ246833	ND5,	NADH-ubiquinone oxidoreductase chain 5	–2.0502
		DQ246833	ND6,	NADH-ubiquinone oxidoreductase chain 6	–2.0502
**hsa04270:Vascular smooth muscle contraction**	0.026411199	NM_001014797	KCNMA1,	potassium large conductance calcium-activated channel, subfamily M, alpha member 1	2.45
		NM_000778	CYP4A11,	cytochrome P450, family 4, subfamily A, polypeptide 11	2.44
		NM_030662	MAP2K2,	mitogen-activated protein kinase kinase 2 pseudogene; mitogen-activated protein kinase kinase 2	2.3426
		NM_001654	ARAF,	v-raf murine sarcoma 3611 viral oncogene homolog	2.33
		AB002378	ARHGEF11,	Rho guanine nucleotide exchange factor (GEF) 11	2.1961
		NM_000678	ADRA1D,	adrenergic, alpha-1D-, receptor	1.94
		NM_001957	EDNRA,	endothelin receptor type A	1.64
		AL049593	PLCB1,	phospholipase C, beta 1 (phosphoinositide-specific)	1.64
		NM_000928	PLA2G1B,	phospholipase A2, group IB (pancreas)	1.61
		NM_182493	MYLK3,	myosin light chain kinase 3	–1.64
**hsa05110:Vibrio cholerae infection**	0.061033032	NM_013336	SEC61A1,	Sec61 alpha 1 subunit (S. cerevisiae)	2.66
		NM_002457	MUC2,	mucin 2, oligomeric mucus/gel-forming	1.83
		NM_004231	ATP6V1F,	ATPase, H+ transporting, lysosomal 14 kDa, V1 subunit F	1.7852
		BC001277	KDELR3,	KDEL (Lys-Asp-Glu-Leu) endoplasmic reticulum protein retention receptor 3	1.5844
		AF363578	ATP6V1C1,	ATPase, H+ transporting, lysosomal 42 kDa, V1 subunit C1	–1.647
		NM_018144	SEC61A2,	Sec61 alpha 2 subunit (S. cerevisiae)	–1.68
					
**hsa00071:Fatty acid metabolism**	0.064488936	NM_000098	CPT2,	carnitine palmitoyltransferase 2	4.8121
		NM_000778	CYP4A11,	cytochrome P450, family 4, subfamily A, polypeptide 11	2.44
		M24310	ADH1A, ADH1B, ADH1C,	alcohol dehydrogenase 1B (class I), beta polypeptide; alcohol dehydrogenase 1A (class I), alpha polypeptide; alcohol dehydrogenase 1C (class I), gamma polypeptide	2.4322
		AC011742	HADHA,	hydroxyacyl-Coenzyme A dehydrogenase/3-ketoacyl-Coenzyme A thiolase/enoyl-Coenzyme A hydratase (trifunctional protein), alpha subunit	–1.5984
		NM_000016	ACADM,	acyl-Coenzyme A dehydrogenase, C-4 to C-12 straight chain	–1.6598
**hsa04370:VEGF signaling pathway**	0.065344049	AL590666	SH2D2A,	SH2 domain protein 2A	3.0366
		NM_030662	LOC407835,MAP2K2,	mitogen-activated protein kinase kinase 2 pseudogene; mitogen-activated protein kinase kinase 2	2.3426
		AK128712	PXN,	paxillin	2.25
		NM_000928	PLA2G1B,	phospholipase A2, group IB (pancreas)	1.61
		AC018445	NFATC1,	nuclear factor of activated T-cells, cytoplasmic, calcineurin-dependent 1	1.5477
		BC035404	PTK2,	PTK2 protein tyrosine kinase 2	–1.55
		NM_004555	NFATC3,	nuclear factor of activated T-cells, cytoplasmic, calcineurin-dependent 3	–1.66
**hsa04670:Leukocyte transendothelial migration**	0.080318923	NM_007052	NOX1,	NADPH oxidase 1	3.6
		AK128712	PXN,	paxillin	2.25
		NM_021195	CLDN6, LOC284620,	claudin 6; similar to claudin 6	2.1396
		AC087269	CLDN23,	claudin 23	2.026
		NM_001078	VCAM1,	vascular cell adhesion molecule 1	1.91
		AC002351	MYL2,	myosin, light chain 2, regulatory, cardiac, slow	1.5014
		NM_182848	CLDN10,	claudin 10	–1.54
		BC035404	PTK2,	PTK2 protein tyrosine kinase 2	–1.55
		BC028224	CYBA,	cytochrome b-245, alpha polypeptide	–1.77
**hsa04062:Chemokine signaling pathway**	0.100104809	NM_005633	SOS1,	son of sevenless homolog 1 (Drosophila)	2.52
		BC067368	ARRB2,	arrestin, beta 2	2.5065
		AC091153	CXCL16,	chemokine (C-X-C motif) ligand 16	2.4391
		AK128712	PXN,	paxillin	2.25
		NM_004972	JAK2,	Janus kinase 2	1.9198
		AL049593	PLCB1,	phospholipase C, beta 1 (phosphoinositide-specific)	1.64
		CR601768	RASGRP2,	RAS guanyl releasing protein 2 (calcium and DAG-regulated)	1.6064
		BC033522	RELA,	v-rel reticuloendotheliosis viral oncogene homolog A (avian)	1.53
		AF053356	GNB2,	guanine nucleotide binding protein (G protein), beta polypeptide 2	–1.5457
		BC035404	PTK2,	PTK2 protein tyrosine kinase 2	–1.55
		NM_002991	CCL24,	chemokine (C-C motif) ligand 24	–1.6283
		AC104850	CCR8,	chemokine (C-C motif) receptor 8	–3.656
**hsa04920:Adipocytokine signaling pathway**	0.111181365	BC051731	MAPK10,	mitogen-activated protein kinase 10	2.42
		NM_004972	JAK2,	Janus kinase 2	1.9198
		NM_000594	TNF,	tumor necrosis factor (TNF superfamily, member 2)	1.78
		BC033522	RELA,	v-rel reticuloendotheliosis viral oncogene homolog A (avian)	1.53
		BC000052	PPARA,	peroxisome proliferator-activated receptor alpha	–1.81
		NM_016203	PRKAG2,	protein kinase, AMP-activated, gamma 2 non-catalytic subunit	–2.18
**hsa04012:ErbB signaling pathway**	0.113641534	NM_005633	SOS1,	son of sevenless homolog 1 (Drosophila)	2.52
		BC051731	MAPK10,	mitogen-activated protein kinase 10	2.42
		NM_030662	LOC407835,MAP2K2,	mitogen-activated protein kinase kinase 2 pseudogene; mitogen-activated protein kinase kinase 2	2.3426
		NM_001654	ARAF,	v-raf murine sarcoma 3611 viral oncogene homolog	2.33
		AC010336	MAP2K7,	mitogen-activated protein kinase kinase 7	1.8723
		NM_002578	PAK3,	p21 protein (Cdc42/Rac)-activated kinase 3	1.64
		BC035404	PTK2,	PTK2 protein tyrosine kinase 2	–1.55
**hsa04660:T cell receptor signaling pathway**	0.117257244	NM_030662	LOC407835,MAP2K2,	mitogen-activated protein kinase kinase 2 pseudogene; mitogen-activated protein kinase kinase 2	2.3426
		AC010336	MAP2K7,	mitogen-activated protein kinase kinase 7	1.8723
		NM_005633	SOS1,	son of sevenless homolog 1 (Drosophila)	2.52
		NM_000594	TNF,	tumor necrosis factor (TNF superfamily, member 2)	1.78
		NM_002578	PAK3,	p21 protein (Cdc42/Rac)-activated kinase 3	1.64
		AC018445	NFATC1,	nuclear factor of activated T-cells, cytoplasmic, calcineurin-dependent 1	1.5477
		BC033522	RELA,	v-rel reticuloendotheliosis viral oncogene homolog A (avian)	1.53
		NM_004555	NFATC3,	nuclear factor of activated T-cells, cytoplasmic, calcineurin-dependent 3	–1.66
**hsa04540:Gap junction**	0.123087444	NM_005633	SOS1,	son of sevenless homolog 1 (Drosophila)	2.52
		NM_030662	LOC407835,MAP2K2,	mitogen-activated protein kinase kinase 2 pseudogene; mitogen-activated protein kinase kinase 2	2.3426
		AL049593	PLCB1,	phospholipase C, beta 1 (phosphoinositide-specific)	1.64
		NM_000794	DRD1,	dopamine receptor D1	1.8933
		AP002364	LOC399942,TUBA1B,	hypothetical gene supported by AF081484; NM_006082; tubulin, alpha 1b	–1.5714
		AP003120	GRM5,	glutamate receptor, metabotropic 5	–1.621
		BC008838	MAP2K5,	mitogen-activated protein kinase kinase 5	–1.63
**hsa04514:Cell adhesion molecules (CAMs)**	0.130286072	AY819760	MADCAM1	mucosal vascular addressin cell adhesion molecule 1	2.43
		NM_021195	CLDN6,LOC284620,	claudin 6; similar to claudin 6	2.1396
		NM_018977	NLGN3,	neuroligin 3	2.04
		AC087269	CLDN23,	claudin 23	2.026
		AC009153	GLG1,	golgi apparatus protein 1	1.9457
		NM_001078	VCAM1,	vascular cell adhesion molecule 1	1.91
		NM_182848	CLDN10,	claudin 10	–1.54
		AF099810	NRXN3,	neurexin 3	–1.6057
		BX005428	HLA-1F,	major histocompatibility complex, class I, F	–1.6678
**hsa04810:Regulation of actin cytoskeleton**	0.43	NM_005337	NCKAP1L	NCK-associated protein 1-like	2.8097
		NM_005633	SOS1	son of sevenless homolog 1 (Drosophila)	2.52
		NM_030662	MAP2K2	mitogen-activated protein kinase kinase 2	2.3426
		NM_001654	ARAF	v-raf murine sarcoma 3611 viral oncogene homolog	2.33
		AK128712	PXN	paxillin	2.25
		NM_002578	PAK3	p21 protein (Cdc42/Rac)-activated kinase 3	1.64
		BC035404	PTK2	PTK2 protein tyrosine kinase 2	–1.55
		NM_182493	MYLK	myosin light chain kinase	–1.64
		NM_002009	FGF7	fibroblast growth factor 7 (keratinocyte growth factor)	–1.6473
**hsa04010:MAPK signaling pathway**	0.4343	BC067368	ARRB2	Arrestin, beta 2	2.5065
		NM_030662	MAP2K2	mitogen-activated protein kinase kinase 2	2.3426
		NM_000594	TNF	Tumor necrosis factor (TNF superfamily, member 2)	1.78
		NM_000928	PLA2G1B	Phospholipase A2, group IB (pancreas)	1.61
		BC033522	RELA	V-rel, nuclear factor of kappa B, p65	1.53
		BC008838	MAP2K5	Mitogen-activated protein kinase kinase 5	–1.63

List of key KEGG Pathways extracted from differentially expressed genes utilizing DAVID Bioinformatic Resource. Specific pathways are arranged in order of their p-value. Gene names and Fold changes for individual genes were added from the original data set.

### HAPE-specific Inter Genic Networks

**Figure 5:**
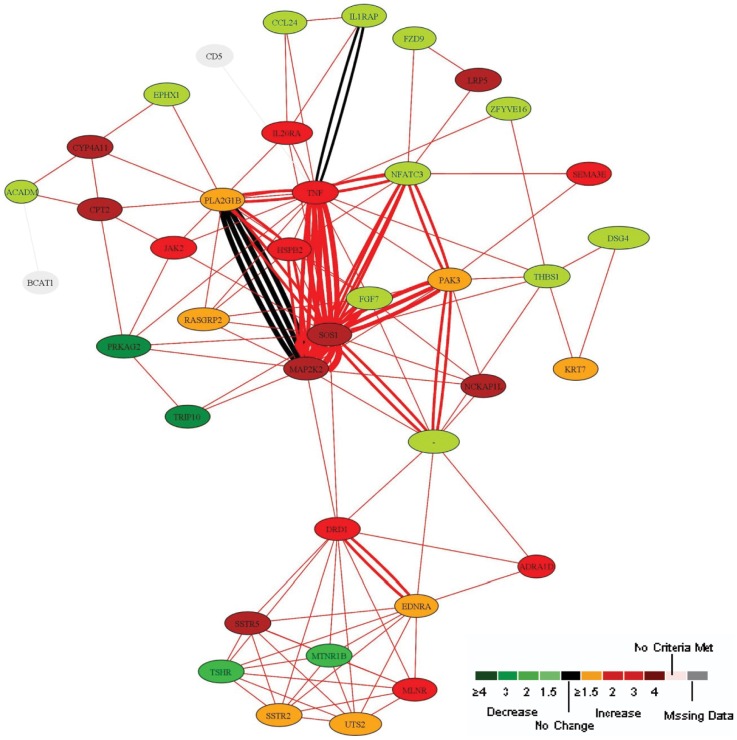
Intergenic Network. The figure represents an integrated network of multiple pathways interacting through common gene products. Specific sub-networks (gene associations within individual pathways) constituting the integrated network are highlighted in Supplementary Figure S3 (A-R). The major sub-network pathways include regulation of actin cytoskeleton, calcium signaling, MAPK pathway, immune cell signaling, cell-cell communication, VEGF signaling amongst others. The network shown was extracted from the list of differentially expressed genes utilizing ‘Pathway Miner’. Relationships between the genes (nodes connected by edges) suggest co-occurrence in a biological pathway. Weight of a specific edge is a relative representation of the number of pathways in which the associating nodes (gene products) co-occur in the KEGG pathway resource. The nodes are labeled by gene names and colored based on the respective fold changes for specific genes in the original data set.

We next utilized Pathway Miner [20] to visualize the interactions between differentially regulated genes. Interacting nodes were extracted at p≤0.05 and represented along with color scheme to indicate the respective fold changes (Figure 5). Specific genes which feature within these pathways along with respective fold changes are represented as Supplementary Table S5. The integrated network contained a hub of common nodes which included *TNF alpha*, *MAP2K2*, *PLA2G1B*, *PAK3*, *SOS1* (Figure 5). These nodes were connected to smaller sub-networks with genes composing specific pathways such as regulation of actin cytoskeleton, MAPK signaling, VEGF signaling, Adipocytokine signaling, T-cell receptor signaling, gap junctions and focal adhesion, fatty acid metabolism amongst others. These sub-networks (specific pathways) as part of the bigger network are illustrated in Supplementary Figure S3A-R.

### Hypoxia responsive gene transcripts

In lines with the ‘Diathesis-Stress models’, we sought to determine if there was any apparent difference in the basic hypoxia responses between HAPE and acclimatized individuals. To this end, we searched for differentially expressed hypoxia responsive transcripts in our data set. Interestingly, we observed several such transcripts in our HAPE dataset (Table 2). Hypoxia inducible proline hydroxylase, egl nine homolog 3 (*EGLN3*), endothelin converting enzyme 1 (*ECE 1*), endothelin receptor type A (*EDNRA*), ADAM metallopeptidase domain 11 (*ADAM 11*), adrenergic receptor *ADRA1D*, angiopoeitin 4 (*ANGPT4*), claudin 6 (*CLDN6*), carbonic anhydrase IX (*CA9*), cytochrome P450 and voltage gated sodium channels (*SCN10A*, *SCN8A*) were upregulated while the expression of angiopoeitin-like 1 (*ANGPTL1*), protein tyrosine kinase 2 (*PTK2*), peroxisome proliferator-activated receptor alpha (*PPARA*), Rho GTPase activating protein 24 (*ARHGAP 24*), thrombospondin 1 (*THBS1*), Heat shock protein 90 (*HSP 90AB3P*, a molecular chaperone), histone deacetylase 9 (*HDAC9*), water channel aquaporin 12 (*AQP12*) and ubiquilin 4 (*UBQLN4*) were downregulated in HAPE compared to acclimatized controls. This data underscores prominent differences in responses of two groups of individuals to hypoxia at high altitude.

**Table 2:** Genes involved in early responses to hypoxia.

Gene Name	Accession No.	Description	Fold Change
ECE1	NM_001397	Endothelin converting enzyme 1	2.8993
SCN8A	NM_014191	Sodium channel, voltage gated, type VIII, alpha subunit	2.7
ADAMTS7	NM_014272	ADAM metallopeptidase with thrombospondin type 1 motif, 7	2.56
CA9	NM_001216	Carbonic anhydrase IX	2.527
CYP4A11	NM_000778	Cytochrome P450, family 4, subfamily A, polypeptide 11	2.44
EGLN3	NM_022073	Egl nine homolog 3 (C. elegans)	2.2093
CLDN6	NM_021195	Claudin 6	2.1396
ADRA1D	NM_000678	Adrenergic, alpha-1D-receptor	1.94
ANGPT4	NM_015985	Angiopoietin 4	1.7871
EDNRA	NM_001957	Endothelin receptor type A	1.64
SCN10A	NM_006514	Sodium channel, voltage-gated, type X, alpha subunit	1.5808
PTK2	BC035404	PTK2 protein tyrosine kinase 2	–1.55
THBS1	NM_003246	Thrombospondin 1	–1.61
ANGPTL1	NM_004673	Angiopoietin-like 1	–1.6608
AQP12A	NM_198998	Aquaporin 12A	–1.9375
ARHGAP24	NM_001025616	Rho GTPase activating protein 24	–2.18
UBQLN4	NM_020131	Ubiquilin 4	–2.51

List of differentially expressed hypoxia-responsive genes in HAPE individuals.

## Discussion

**Figure 6:**
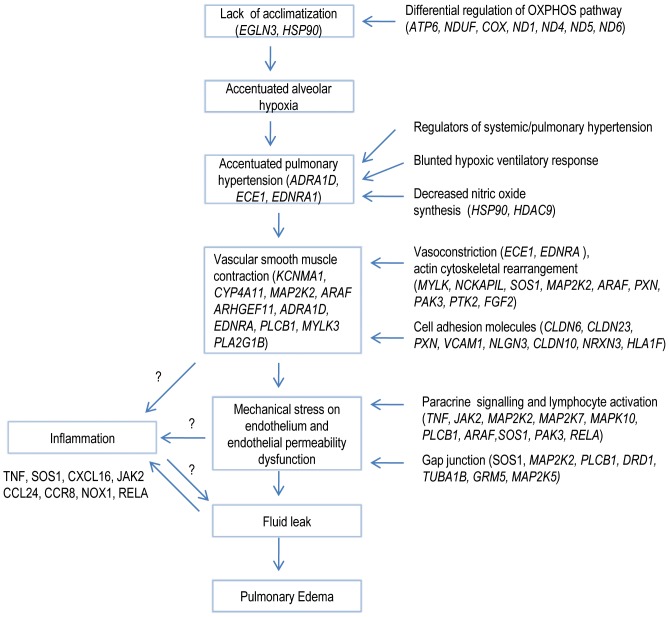
Schematic representation of possible events occurring during HAPE. The pathways suggested by the current data set have been integrated with established phenomenon such as pulmonary vasoconstriction, elevated pulmonary artery pressure which are known to precede HAPE. Perturbation of pathways such as those regulating vasoconstriction, inflammation, gap junctions and adhesion molecules can dysregulate vascular homeostasis leading to fluid leak and edema formation.

There has been an ever increasing body of evidence in favour of ‘stress failure’ being one of the early phenomenon which initiates the cascade of events in HAPE. However, it has also been argued in literature that initial damage to blood-gas barrier because of ‘stress failure’ is self limiting owing to the propensity for quick closure of this barrier after pressure reduction [23]. This strongly suggests the existence of confounding parallel factors complementing initial stress failure for clinical presentation of HAPE which may arise from conjunction of multiple factors besides capillary stress failure [24]. Our data supports this notion and we suggest concurrent modulation of multiple factors which govern vascular homeostasis (balance between vascular injury and vascular repair) and perhaps perturbed lung fluid dynamics during HAPE. Vascular injury is usually induced by factors such as altered shear stress, oxidative stress, cytokine, and vascular tone modulators such as endothelin-1, angiotensin II amongst others**.** Notwithstanding the limitation of single time point study which is less likely to suggest cause/consequence relationship between various events, our global expression profiling for HAPE and acclimatized individuals evoked several interesting mechanistic possibilites besides generating molecular evidence for well known physiological processes involved in HAPE [23], [25]. We observed multiple pathways to be modulated under these conditions which appear to be reminiscent of three broad physiological processes: 1) regulation of capillary pressure (stress failure), 2) modulation of barrier function (endothelial permeability/dysfunction) and 3) differential regulation of hypoxia sensing/response pathways. Pathways which could directly regulate capillary pressure included smooth muscle contraction, cellular actin cytoskeleton rearrangements and regulation of systemic/pulmonary hypertension. Modulation of paracrine signaling through chemokines, lymphocyte activation and modulation of junctional proteins could potentially regulate endothelial permeability and dysfunction. Notably, under conditions of perturbed endothelial permeability, HAPE can occur at much lower capillary pressure [25], [26]. Lastly, differential regulation of metabolic pathways (OXPHOS, Fatty acid metablism), VEGF signaling and specific individual genes such as EGLN3 suggest significant differences in hypoxia sensing/responses between acclimatized and HAPE individuals. Specific gene clusters, pathways and other individually curated genes from our dataset which support our proposition of perturbed vascular homeostasis through above mentioned pathways are discussed below and depicted schematically in Figure 6.

### 1) Pathways Regulating Capillary Stress Failure

#### Vasoconstriction and actin cytoskeletal organisation.

Pulmonary capillary ‘stress failure’ is described as the mechanical breakdown of basement membrane and pulmonary capillary endothelium resulting from excessive intravascular pressure [6], [27], [28]. This increase in intravascular pressure is a likely consequence of vasoconstriction at tissue level (blood vessels) regulated further by actin cytoskeletal reorganization at cellular level (vascular smooth muscle cells). Our data set revealed several transcripts which could be classified as potential regulators of vascular smooth muscle contraction (hsa:04270) (Table 1). Furthermore, gene transcripts associated with regulation of actin cytoskeleton namely *PAK3, MAP2K2, SOS1, ARAF, NCKAP1L, PXN,* (KEGG_pathway hsa:04810) were up regulated in HAPE (Table 1) though *PTK2, MYLK3* and *FGF7* were down regulated. Several other transcripts associated with actin cytoskeleton components like *ACTR2*, *SVIL, SPTBN1, SLC9A3R1, TNNI1* and *MYO5C* were also up regulated in individuals with HAPE. Notably, we observed *ARHGAP24* which encode negative regulators of Rho GTPases and are implicated in actin remodeling, cell polarity and cell migration [29] to be down regulated in our data set. Hypoxia *per se* is known to culminate in vasoconstriction. Further, we too have observed modulation of vascular smooth muscle contraction (has:04270) and actin cytoskeleton regulation (has:04810) in healthy volunteers on ascent to high altitude during process of acclimatization [30]. Taken together, HAPE-selective marked over-representation of genes regulating vasoconstriction and actin cytoskeletal rearrangement pathways, as seen in the present data set, is a possible evidence for ‘exaggerated vasoconstriction’ – an important factors which is likely to perturb pressure gradient across pulmonary capillaries.

#### Endothelin converting enzyme, endothelin receptor and hypoxic pulmonary vasoconstriction.

In the present study, we observed several upstream regulators of vasoconstriction in HAPE compared to the acclimatized controls; one of them being upregulated transcript of endothelin converting enzyme (*ECE 1*). *ECE 1* generates endothelin-1 (ET-1) from its inactive intermediate and its expression levels have been reported to increase in mouse carotid body under physiological hypoxia [31]. Increased lung endothelin content contributing to lung water accumulation and promoting development of pulmonary edema has been reported in young rats exposed to moderate environmental hypoxia [32], [33]. An augmented release of endothelin-1 and/or its reduced pulmonary clearance in HAPE-susceptible mountaineers was hypothesized to represent one of the mechanisms contributing to exaggerated pulmonary hypertension at high altitude [34]. Increased level of endothelin 1 [34], [35] and decreased availability of nitric oxide [36], [15] have emerged as strong mediators of HPV in HAPE. Our observation of upregulated expression of *ECE1* in HAPE would logically imply higher production of ET-1 [37] consequently culminating in exaggerated HPV in such individuals.

Besides *ECE 1*, upregulated transcripts of endothelin receptor type A (*EDNRA*) were also observed in HAPE individuals. Endothelin receptor type A plays an important role in hypertension, vascular remodeling, cardiac hypertrophy and coronary artery disease [38] and is found on pulmonary vascular smooth muscle cells and adventitial fibroblasts. EDNRA antagonist *in vivo* has been shown to inhibit HPV [39], [40] and also ameliorates edema formation through decreased leakage of fluid from vasculature into the air spaces [32]. In view of functionally upstream regulation of key physiological pathways by *EDNRA* and *ECE 1*, we speculate these genes to be key nodes in pathways regulating elevated pulmonary artery pressure in HAPE. These genes may act as potential genetic markers of high altitude acclimatization/maladaptation and these genomic loci should be scanned for identifying differences in HAPE and acclimatized individuals.

#### Regulation of systemic blood pressure in HAPE.

We observed up regulated expression of adrenergic alpha-1D receptor (*ADRA1D*), which is potentially involved in regulation of systemic blood pressure, to be upregulated in HAPE. Adrenergic receptors mediate sympathetic responses such as smooth muscle contraction causing vasoconstriction in blood vessels including arteries to heart [41], renal artery [42] as well as smooth muscle contraction of bronchioles. *ADRA1D* is one of the three subtypes of the α1 adrenergic receptor family that act as primary catecholamine receptor in the lungs. Enhanced α1 adrenergic trophic activity has been reported in hypoxic pulmonary hypertensive rats [43]. Expression studies from healthy human have shown all the three α1 receptor subtypes to be present in peripheral blood lymphocytes with expression of α1D being the least [44]. Considering that HAPE is characterized by distinctly increased pulmonary artery pressure preceding edema formation [1], upregulated *ADRA1D* along with other upregulated gene transcripts of *ECE1, CYP11B2, PCSK5* and *EDNRA*, as observed in our data set, suggests cumulative role of multiple genes in elevating pulmonary arterial blood pressure thereby supporting multi-factorial dependence for clinical presentation of HAPE.

#### Heat shock protein (HSP) 90 and nitric oxide production in HAPE.

Another important observation in HAPE individuals was the downregulation of heat shock protein 90 kDa (*HSP90AB3P*) suggesting that many important signaling pathways may be compromised in these individuals. HSP90 is involved in HIF signaling pathway as well as many other important cellular events including protection of vital proteins of stress signal transduction pathway. Through altered binding of HSP90, activation state of nitric oxide synthase type 3 (*NOS3*) and nitric oxide (NO) formation was shown to be crucially regulated by polymerization state of beta actin in human platelets [45]. Studies have shown decreased NO concentration in exhaled air in HAPE susceptible individuals during hypoxic exposure at low altitude [16] and during development of HAPE at high altitude [15]. Further, lower concentration of nitrate and nitrite in broncho-alveolar lavage fluid was observed in mountaineers who developed HAPE compared to the controls [46]. It is plausible that decreased expression of *HSP90* in HAPE, as seen in present study, may inhibit tertiary complex formation between NOS3, beta actin and HSP90 thereby resulting in decreased NO formation in HAPE.

Another intriguing observation was down regulation of histone deacetylase 9 (*HDAC9*) in HAPE. HDAC directly regulates HSP90 function for nuclear receptor activity [47]. Considering that both *HSP90* and *HDAC9* transcripts were downregulated in HAPE, the mechanism of action of these regulators and their significance in cellular adaptation to high altitude hypoxia should be probed further.

### 2) Regulation of Barrier function in HAPE.

#### Leukocyte activation, inflammation and adhesion molecules.

The gene expression data obtained in the present study also showed modulation of chemokine signalling pathway, T-cell receptor signalling pathway, leukocyte endothelial transmigration, adhesion and GAP junction molecules in circulating blood cells (Table 1). Notably, studies with lavage fluid from HAPE individuals have yielded conflicting evidence for the involvement of inflammation during early HAPE: while some studies have reported presence of pro-inflammatory cytokines in BAL fluid [48]–[51] others have not observed the same [46]. Interestingly, the present study showed up regulation of transcripts related to inflammation in the blood milieu of HAPE individuals. We raise a possibility that these gene products may engage in a positive feed forward loop pertaining to endothelial permeability/dysfunction and fluid leak (Figure 6). Activation of such pathways will be capable of perturbing endothelial barrier function through multiple levels – directly through the altered expression of GAP junctions or indirectly through endothelial activation in response to lymphocyte-endothelial interactions and inflammatory molecules. It is also possible that such pathways can function cooperatively with initial vasoconstriction and stress failure to skew vascular homeostasis towards leakage phenotype observed in HAPE. In this context our observation of up regulated expression of tumor necrosis factor (*TNF*) in individuals with HAPE is also interesting. Our observation is in line with recent demonstration of increased TNF alpha along with IL-6, VEGF and NO in serum of HAPE patients [48]. Furthermore, studies have also shown that TNF alpha at high altitude increases lung permeability [49], [50], [51]. Our observation of increased expression of TNF could imply plausible role of this transcript in active modulation of lung permeability in HAPE.

### 3) Regulation of Hypoxia sensing/response pathways

#### Metabolic Pathways.

Several genes implicated in oxidative phosphorylation (OXPHOS) (hsa:00190) and fatty acid metabolism (has:00071) were also observed to be differentially expressed in HAPE. At basic cellular level, hypoxia responses are known to culminate in dynamic metabolic adaptation mediated by HIF1α stabilization: up regulation of glycolic pathway genes and modulation of cytochrome oxidase subunit genes [52]. In view of this information, our observation of differential regulation of OXPHOS pathway genes in individuals with HAPE compared to acclimatized individuals was particularly intriguing and to the best of our knowledge, no such suggestion is available in literature. Differential regulation of OXPHOS pathway can manifest varied physiological responses. While one of the possible mechanisms through which OXPHOS pathways regulate HAPE could be unsustainable increase in oxidative stress; it is also possible that differential regulation of the OXPHOS genes may directly regulate vulnerability to HAPE through metabolic route and steady state ATP dynamics. It is tempting to speculate that specific polymorphisms associated with these genes may regulate individual susceptibility to HAPE.

#### Early response to hypoxia.

Another interesting observation in our study was differential expression of egl nine homolog 3 (*EGLN3)* which was upregulated 2.2 fold in HAPE compared to the acclimatized individuals (controls). We also observed near 1.5 fold change in *EGLN1* transcript in HAPE although with insignificant p-value (0.1) (data not shown). EGLNs are a member of hypoxia inducible proline hydroxylases (PHD) family which function as cellular oxygen sensors and directly regulate HIF-α subunit stability [53] thereby regulating cellular response to oxygen availability. Under normoxic condition, EGLNs target HIF for proteosomal degradation through hydroxylation while under hypoxic condition, hydroxylation is significantly decreased leading to stabilization of HIF-α subunits [54]. EGLN isoforms show distinct patterns of expression in various cell types: while *EGLN2* (PHD1) mRNA levels are unchanged or decreased by hypoxia, levels of *EGLN1* (PHD2) and *EGLN3* (PHD3) are increased by hypoxia [55]. PHD3 (EGLN3) has been suggested to contribute more substantially to the regulation of HIF-2α than HIF-1α [55]. Increased expression of *EGLN3* transcript during HAPE in our study and in every likelihood its increased activity implies potential inhibition of HIF responsive element (HRE)-containing genes which are selectively regulated by HIF-2α and thereby modulating basic adaptive response to hypoxia in these individuals. HAPE-specific differential expression of EGLNs observed in our data thus underscores marked differences in hypoxia sensing ability of susceptible individuals and perhaps differential activation of HIF system during high altitude responses. Notably, we did not observe statistically significant difference in the expression of HIF and von Hippel-Lindau tumor suppressor (VHL) transcripts between HAPE and acclimatized individuals.

## Limitations of the study

We understand and note certain limitations of our study. Arguably, the tissue- and temporal-specificity of pathways is not clearly evident from the present data set. Pathogenesis of HAPE is likely to involve modulation of pathways at multiple tissue levels such as pulmonary vasculature and lung epithelium amongst others. An important challenge, therefore, would be to ascribe and validate tissue-specificity of these pathways. Studies with endothelium and/or pulmonary vascular smooth muscles from individuals with HAPE can be helpful in this regard. Furthermore, these pathways are likely to be involved in a temporal and ordered manner in specific cell types. Mechanistic understanding of such complex regulation will certainly benefit from future studies with samples collected pre- and post-induction to high altitude from same individuals.

## Concluding remark

The present study is one of the first global expression profiling in individuals who, after being air lifted to high altitude, succumbed to altitude stress and developed HAPE despite undertaking acclimatization schedule. Given a shear dearth of information on molecular aspects in this area, the current study takes our understanding a step ahead by revealing specific pathways in the complex molecular circuitry of HAPE. Despite certain limitations, our data presented here yields an unambiguous evidence of the multigenic nature of this condition and concurrent modulation of multiple pathways which may regulate vascular homeostasis and lung fluid dynamics. It is probable that individuals who succumb to HAPE on altitude exposure may have genomic sequence alterations, transcripts of which manifest when such individuals are exposed to the negative stress of high altitude. Approaches such as genome wide association and exome sequencing can therefore further enhance our understanding of this altitude illness

## Supporting Information

Figure S1 **Volcano Plot of microarray data.**
(PPTX)

Figure S2 **Graphical Representation of (a) enriched cellular components, molecular functions and biological processes related to the differentially expressed genes in microarray data set.** (b) enriched Gene ontology terms.
(PPTX)

Figure S3 **(A-R): Representation of sub-networks within the integrated network shown in** **Figure 5** **.** Individual pathways (sub-networks) interacting through common nodes generate the integrated network represented in Figure 5. Majority of these sub-networks directly link to the three broad physiological processes described in the manuscript.
(PPTX)

Table S1 **Details of subjects, sample collection and clinical parameters.**
(XLSX)

Table S2 **Minimum Information About A Microarray Experiment (MIAME) compliance of the experimental design, sampling, hybridization and data analysis.**
(DOC)

Table S3 **List of all up and down regulated genes in the microarray data.**
(XLSX)

Table S4 **Functional Annotation Clustering Table (extracted from DAVID Bioinformatic Resource).**
(XLSX)

Table S5 **KEGG, Biocarta and Panther Pathways extracted from Pathway Miner.**
(XLS)
