# Supplementary figures and images for: Genome Wide Expression Analysis Suggests Perturbation of Vascular Homeostasis during High Altitude Pulmonary Edema
Source: PLoS One. 2014 Jan 22;9(1):e85902. doi: 10.1371/journal.pone.0085902 (PMC3899118; doi:10.1371/journal.pone.0085902)

## Slide 1
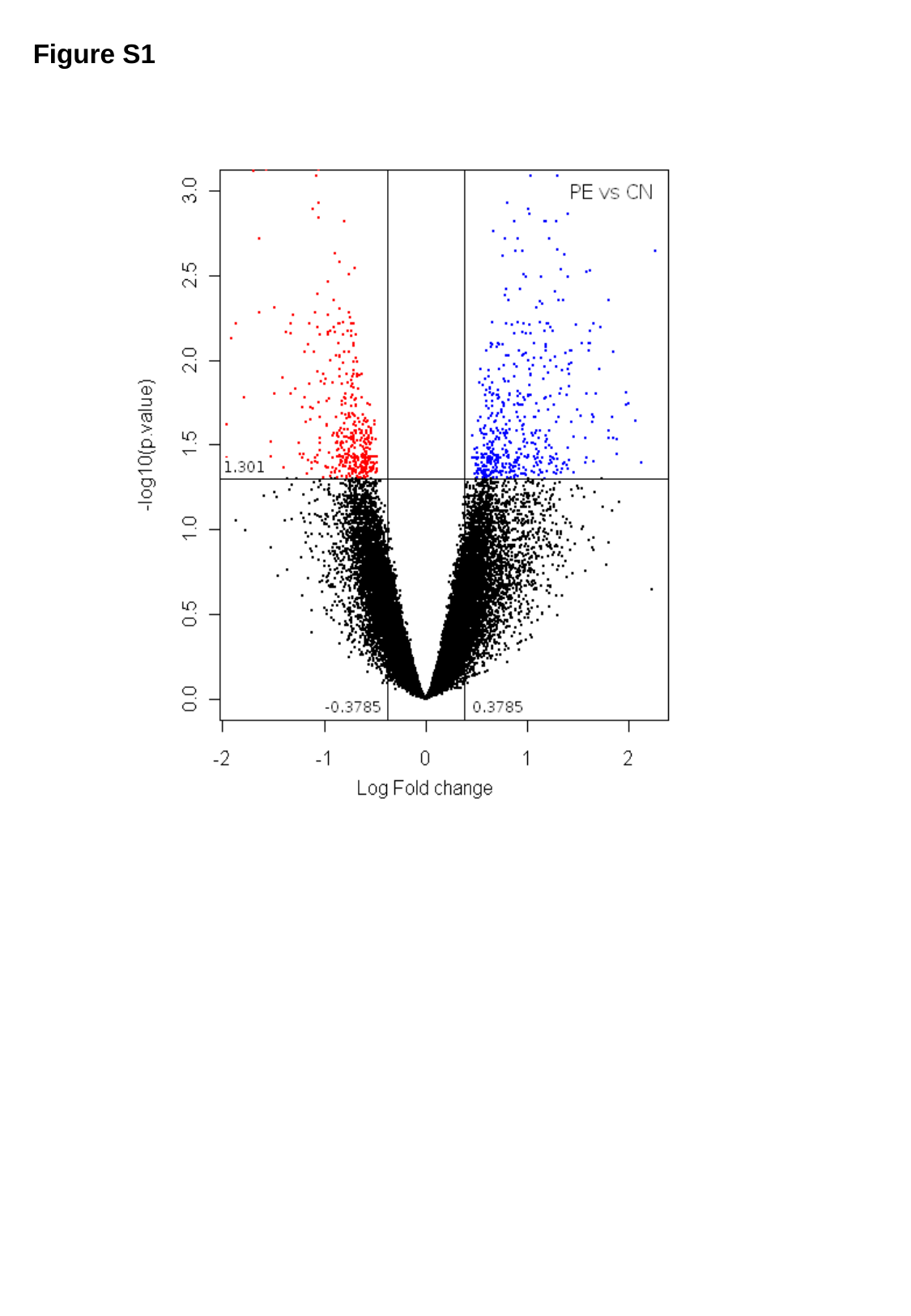

Figure S1

Supplement: Figure S1 — Volcano Plot of microarray data. (PPTX) [file pone.0085902.s001.pptx]
