# Supplementary material for: Genome Wide Expression Analysis Suggests Perturbation of Vascular Homeostasis during High Altitude Pulmonary Edema
Source: PLoS One. 2014 Jan 22;9(1):e85902. doi: 10.1371/journal.pone.0085902 (PMC3899118; doi:10.1371/journal.pone.0085902)
Supplement: Figure S2 — Graphical Representation of (a) enriched cellular components, molecular functions and biological processes related to the differentially expressed genes in microarray data set. (b) enriched Gene ontology terms. (PPTX) [file pone.0085902.s002.pptx]

## Slide 1
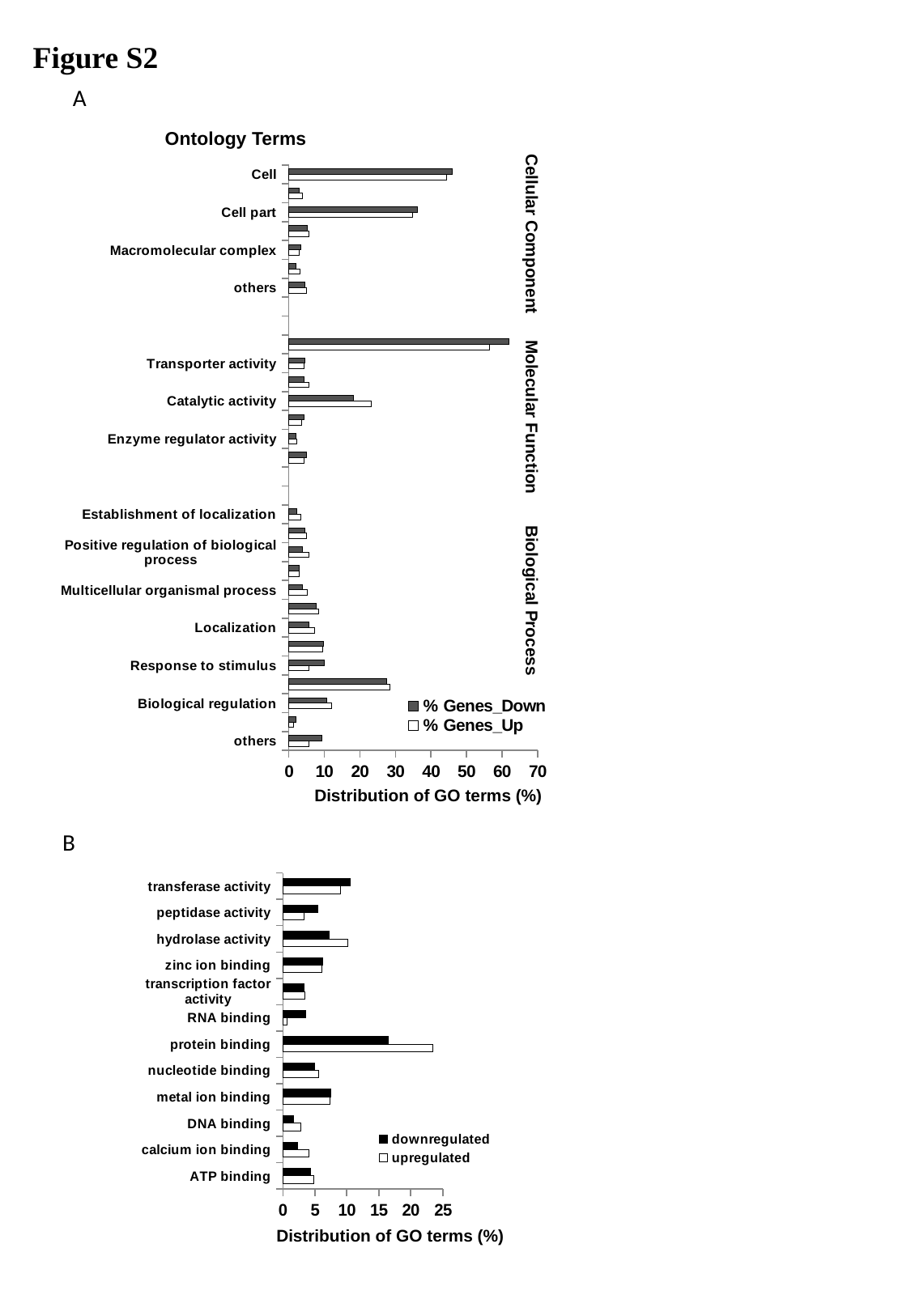

Figure S2
A
Ontology Terms
[unsupported chart]
Cellular Component
Molecular Function
Biological Process
Distribution of GO terms (%)
B
### Chart
| Category | upregulated | downregulated |
|---|---|---|
| ATP binding | 4.81 | 4.27 |
| calcium ion binding | 4.07 | 2.3 |
| DNA binding | 2.77 | 1.6400000000000001 |
| metal ion binding | 7.4 | 7.56 |
| nucleotide binding | 5.55 | 4.930000000000002 |
| protein binding | 23.51 | 16.439999999999987 |
| RNA binding | 0.7000000000000006 | 3.61 |
| transcription factor activity | 3.51 | 3.2800000000000002 |
| zinc ion binding | 6.1099999999999985 | 6.25 |
| hydrolase activity | 10.11 | 7.23 |
| peptidase activity | 3.3699999999999997 | 5.42 |
| transferase activity | 8.98 | 10.59 |Distribution of GO terms (%)
