# Supplementary material for: Genome Wide Expression Analysis Suggests Perturbation of Vascular Homeostasis during High Altitude Pulmonary Edema
Source: PLoS One. 2014 Jan 22;9(1):e85902. doi: 10.1371/journal.pone.0085902 (PMC3899118; doi:10.1371/journal.pone.0085902)
Supplement: Figure S3 — (A-R): Representation of sub-networks within the integrated network shown in Figure 5 . Individual pathways (sub-networks) interacting through common nodes generate the integrated network represented in Figure 5. Majority of these sub-networks directly link to the three broad physiological processes described in the manuscript. (PPTX) [file pone.0085902.s003.pptx]

## Slide 1
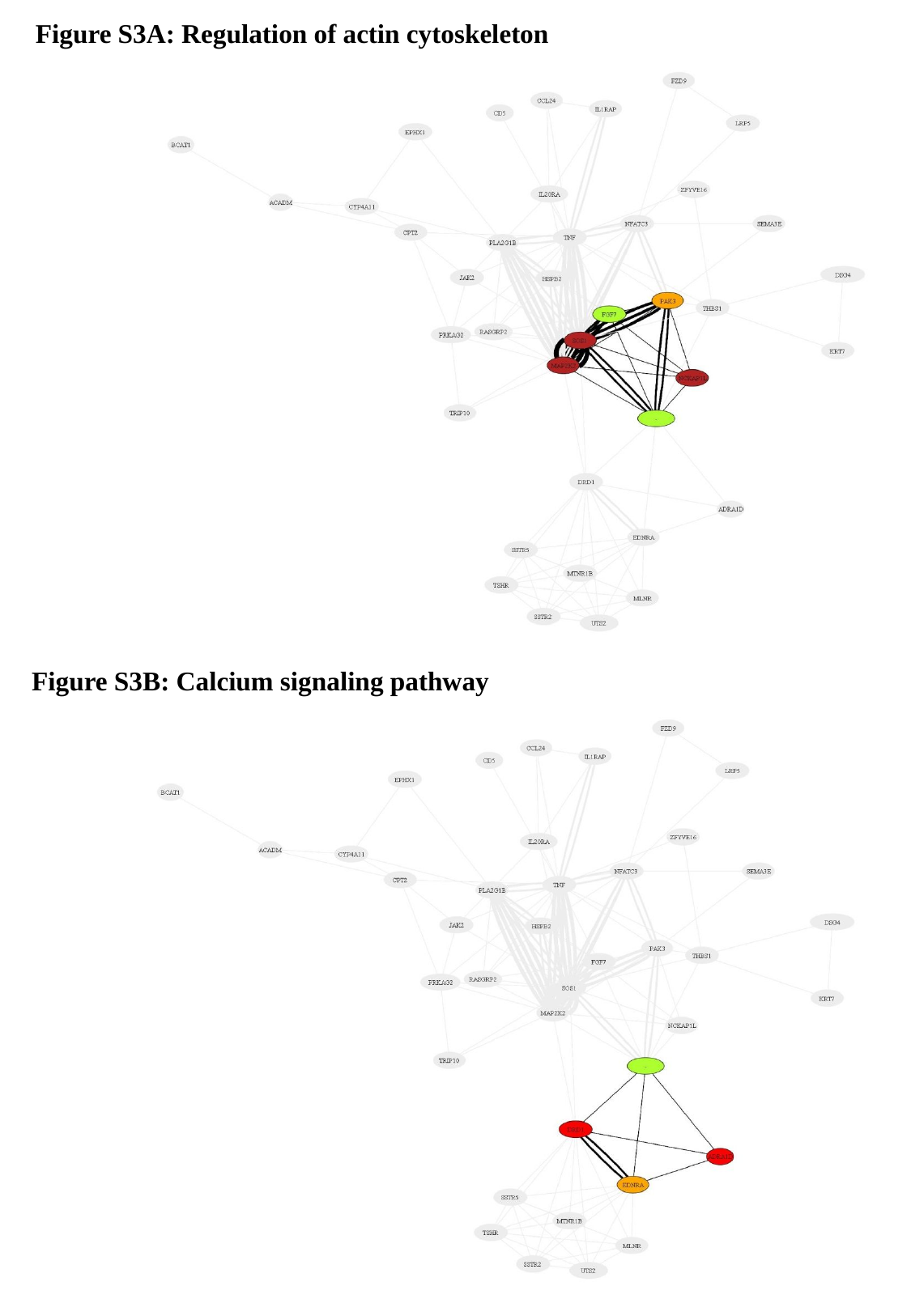

Figure S3A: Regulation of actin cytoskeleton
Figure S3B: Calcium signaling pathway

## Slide 2
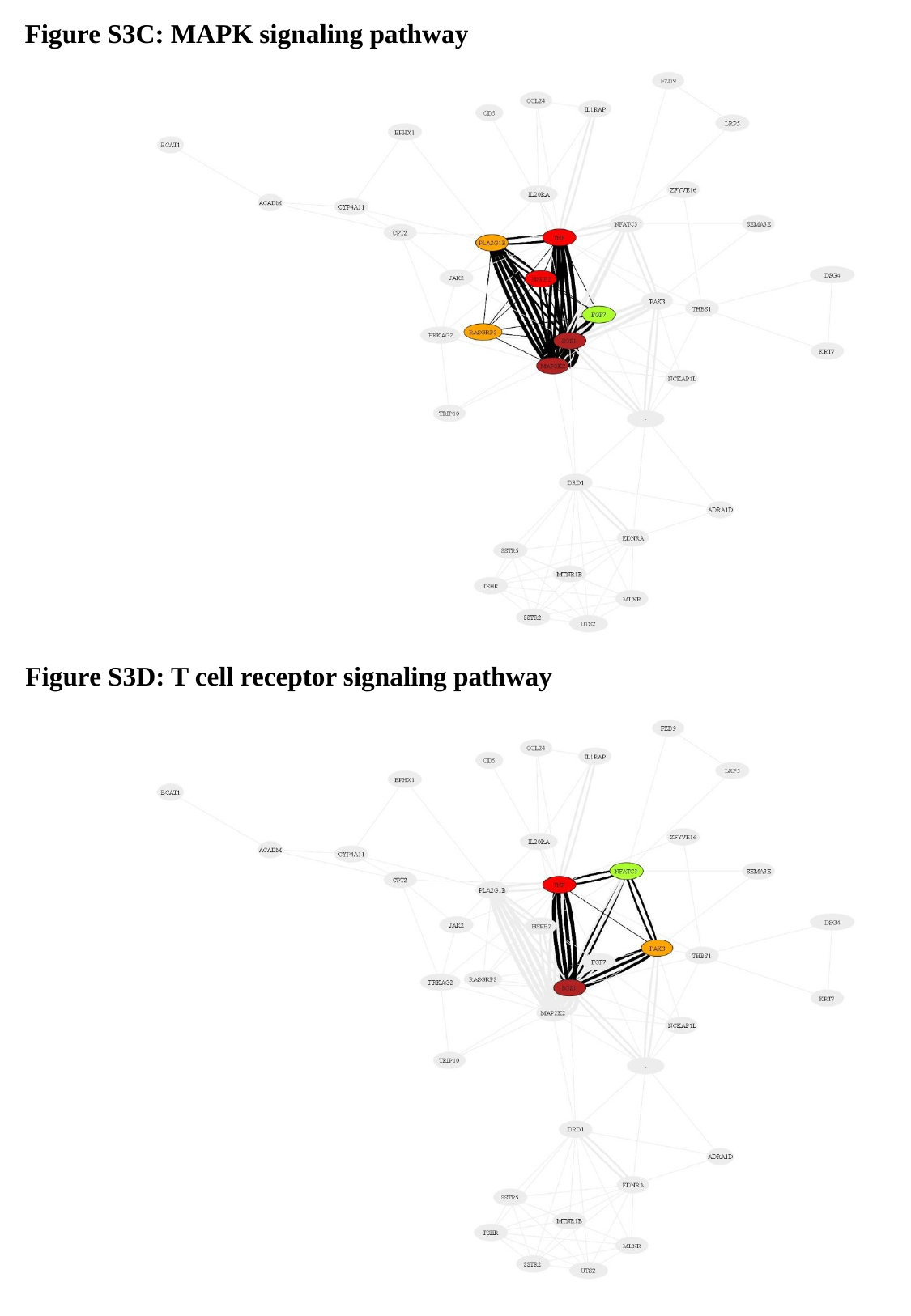

Figure S3C: MAPK signaling pathway
Figure S3D: T cell receptor signaling pathway

## Slide 3
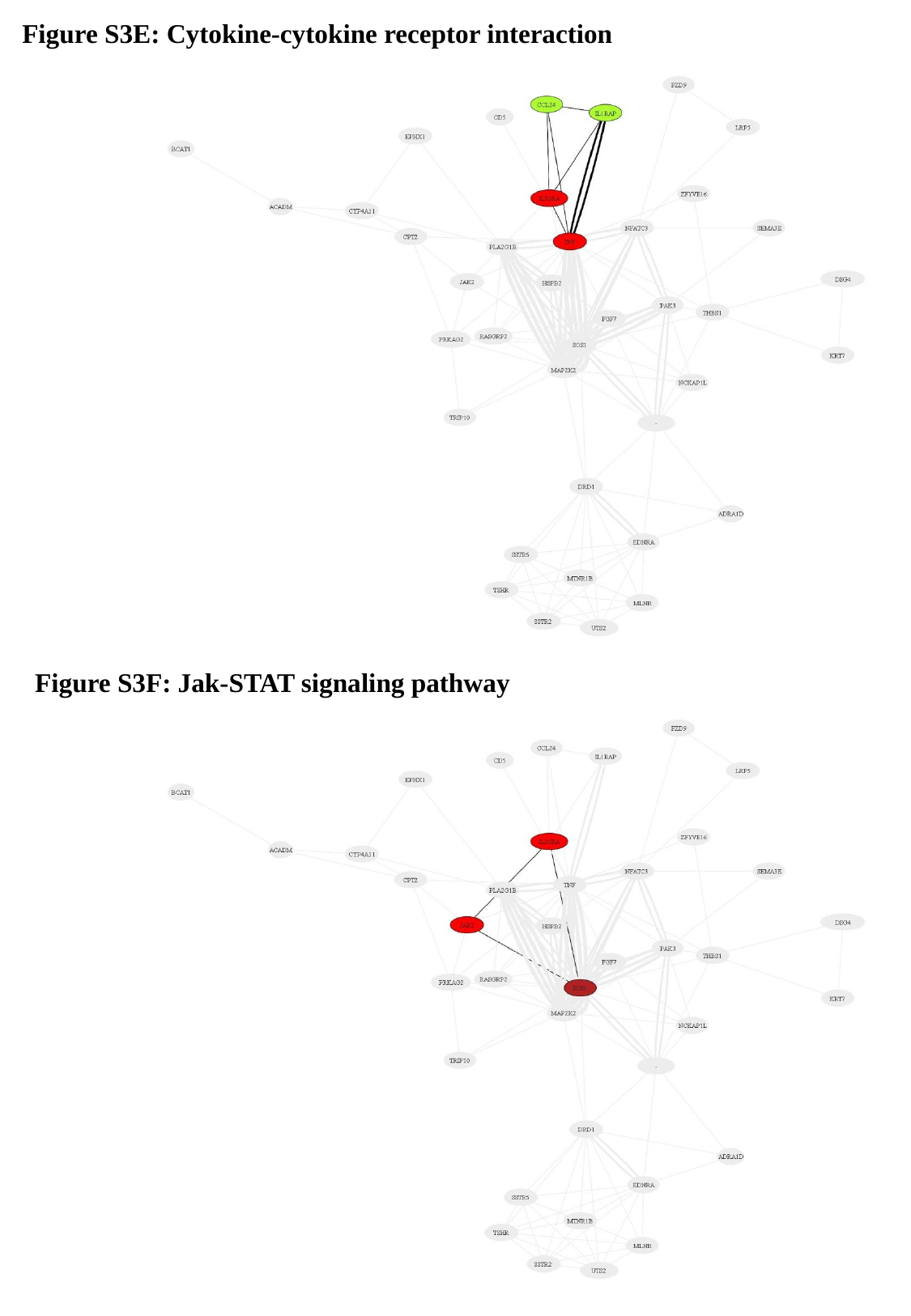

Figure S3E: Cytokine-cytokine receptor interaction
Figure S3F: Jak-STAT signaling pathway

## Slide 4
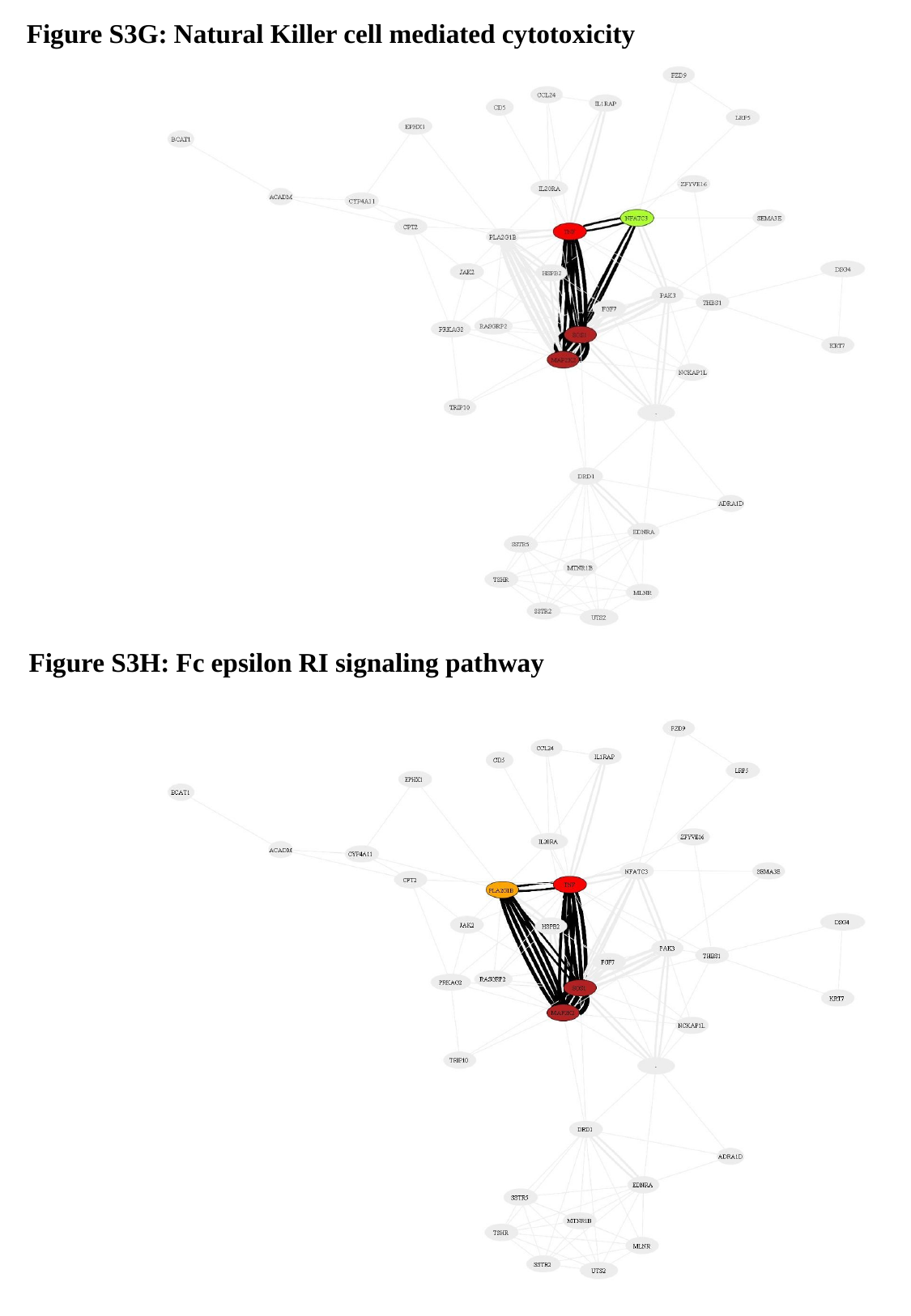

Figure S3G: Natural Killer cell mediated cytotoxicity
Figure S3H: Fc epsilon RI signaling pathway

## Slide 5
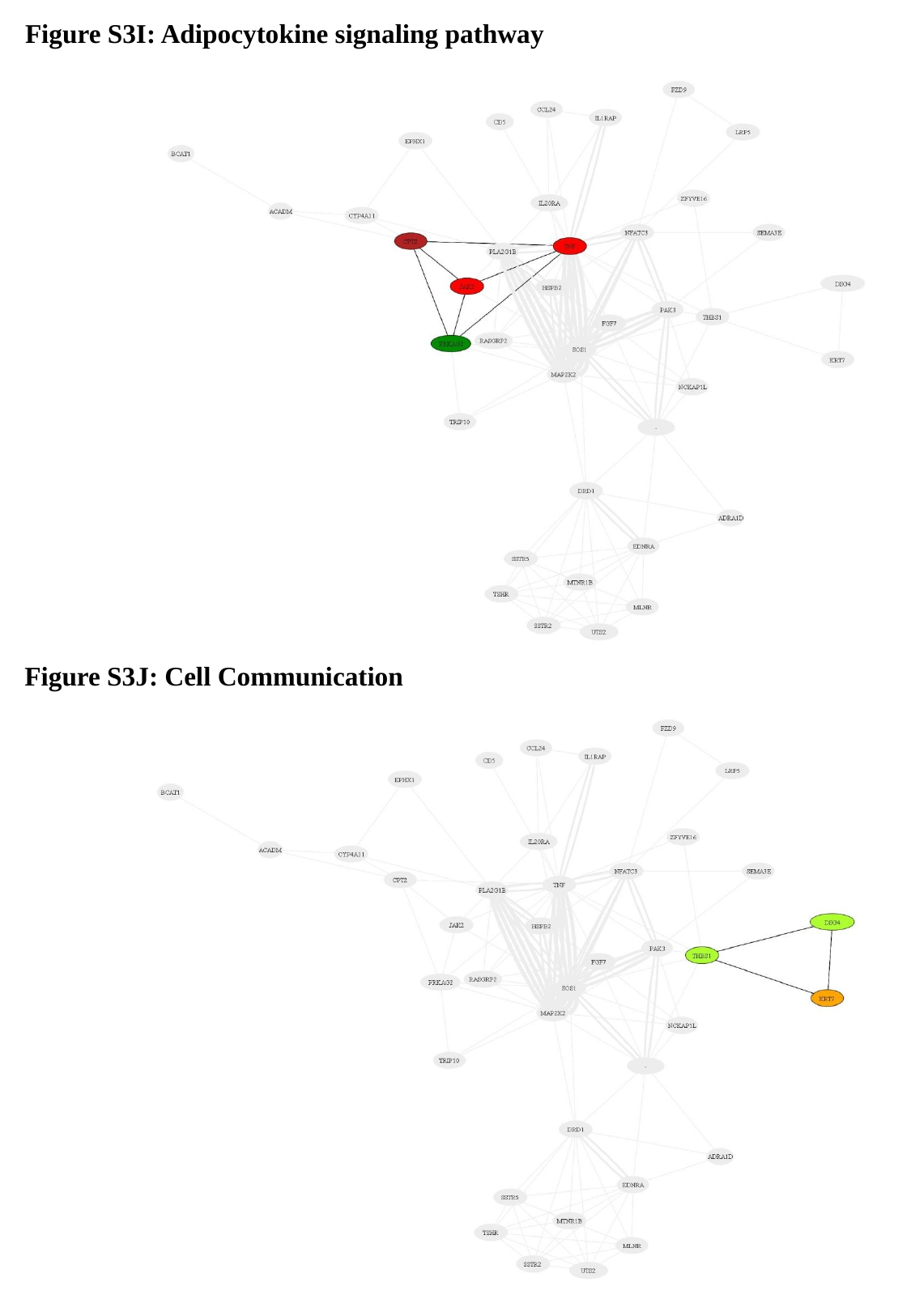

Figure S3I: Adipocytokine signaling pathway
Figure S3J: Cell Communication

## Slide 6
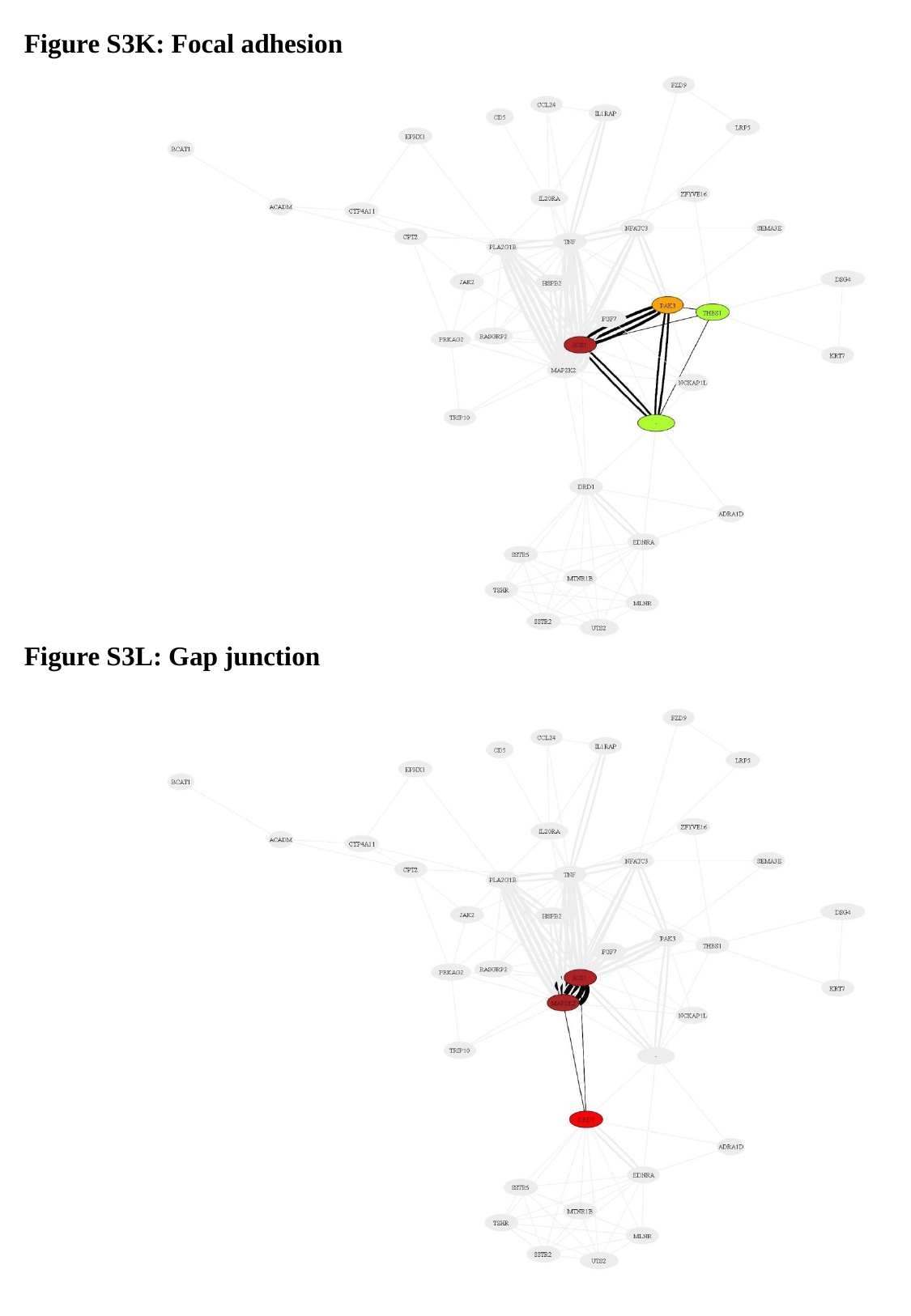

Figure S3K: Focal adhesion
Figure S3L: Gap junction

## Slide 7
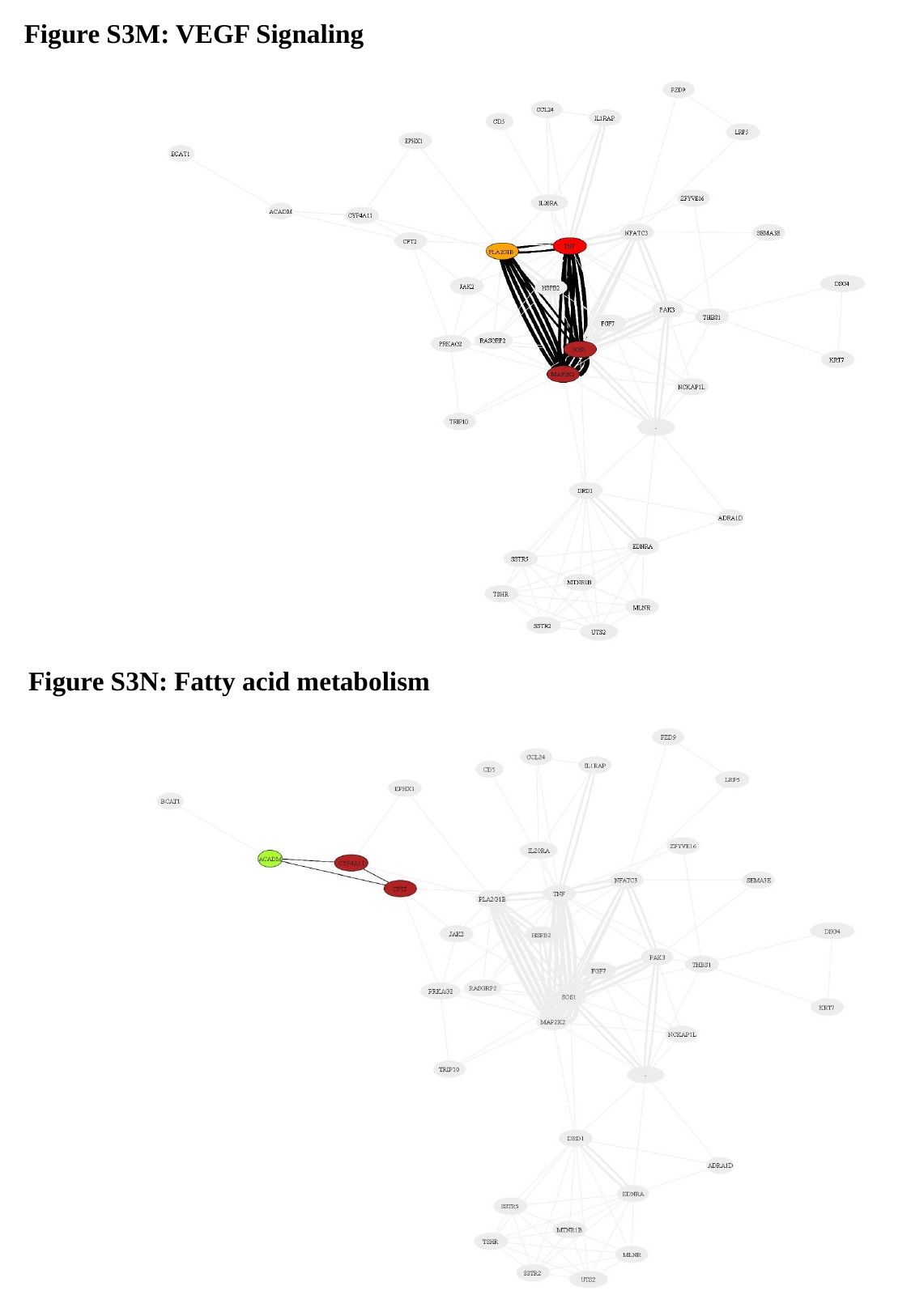

Figure S3M: VEGF Signaling
Figure S3N: Fatty acid metabolism

## Slide 8
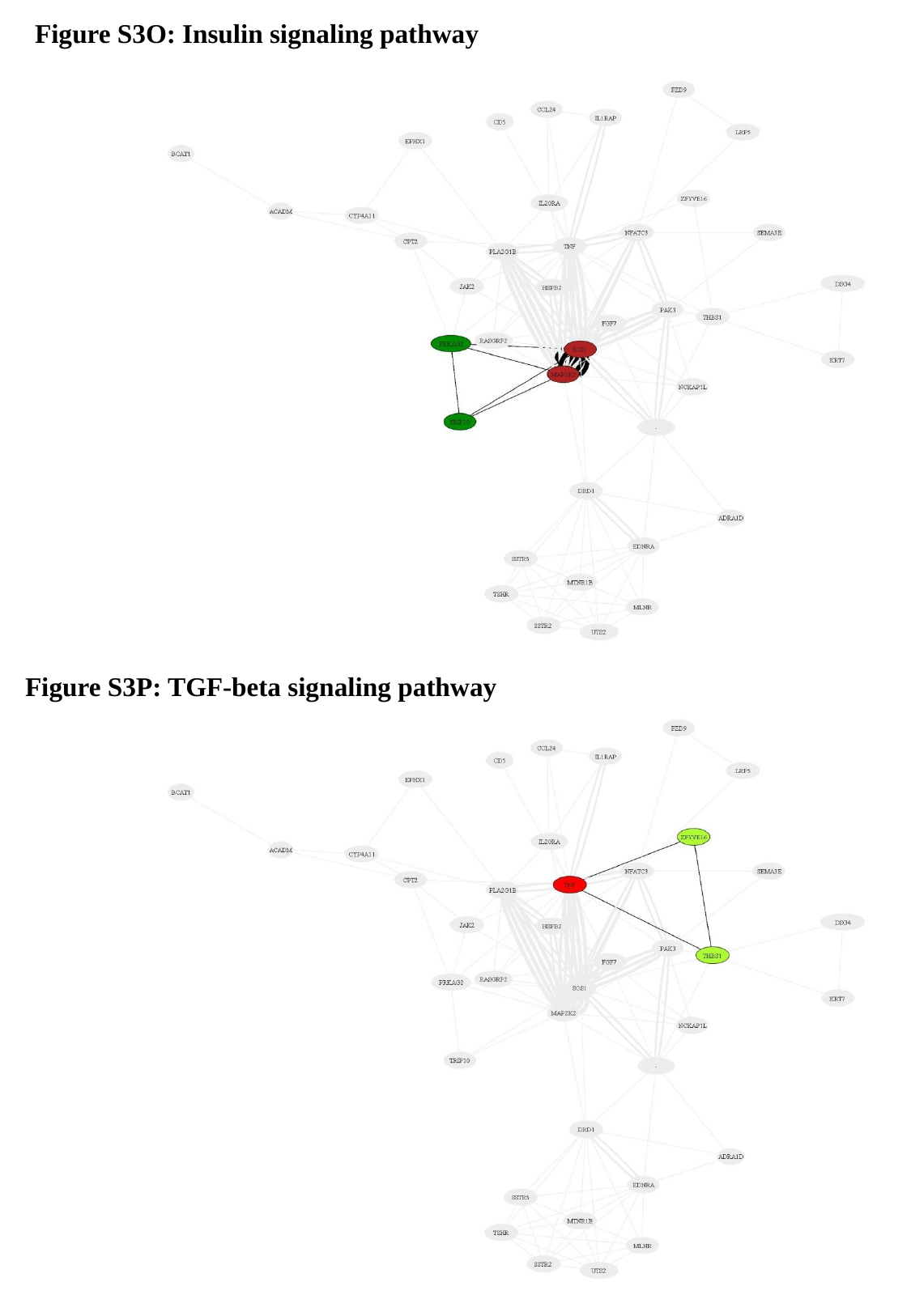

Figure S3O: Insulin signaling pathway
Figure S3P: TGF-beta signaling pathway

## Slide 9
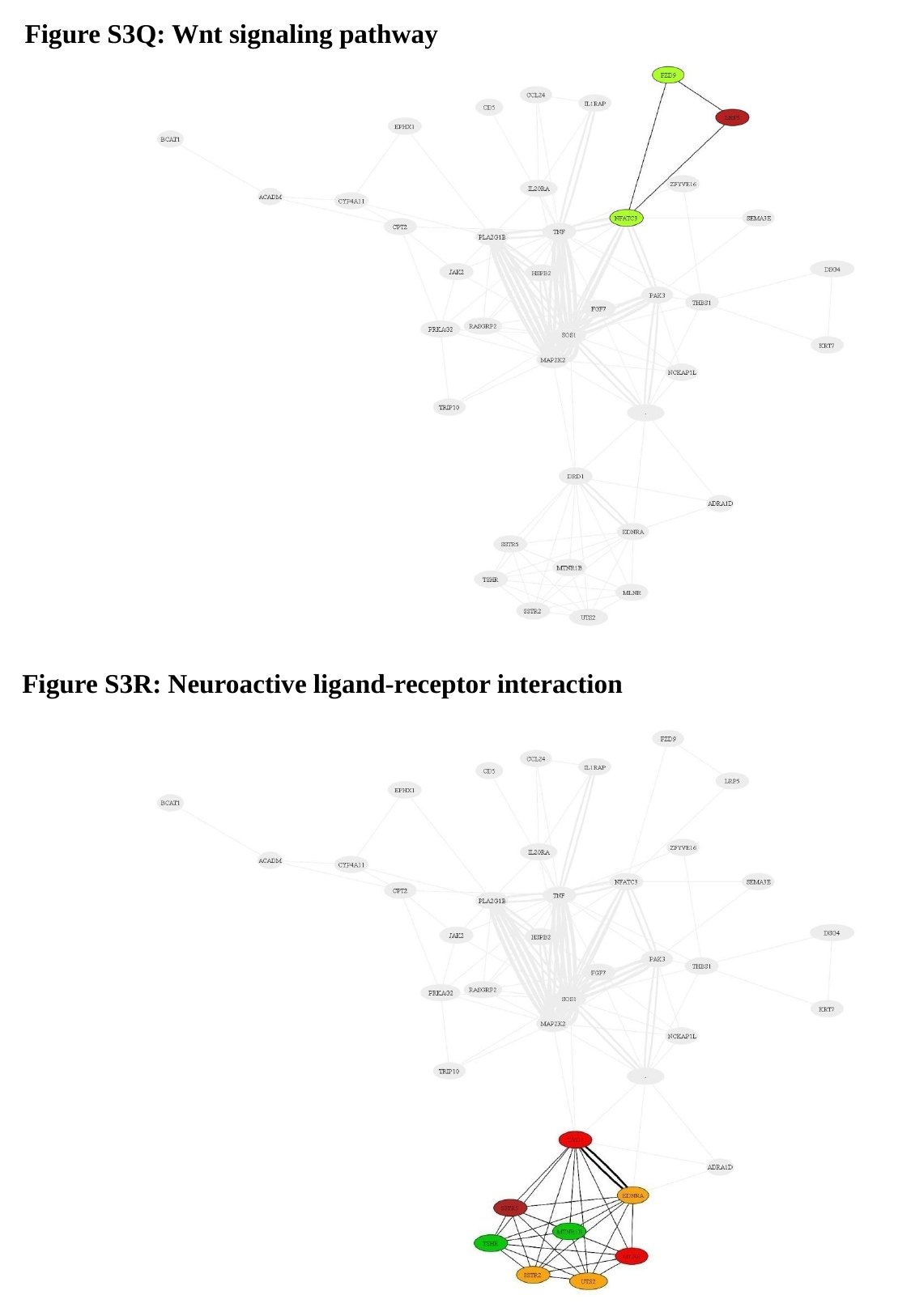

Figure S3Q: Wnt signaling pathway
Figure S3R: Neuroactive ligand-receptor interaction
