# Supplementary material for: Genome Wide Expression Analysis Suggests Perturbation of Vascular Homeostasis during High Altitude Pulmonary Edema
Source: PLoS One. 2014 Jan 22;9(1):e85902. doi: 10.1371/journal.pone.0085902 (PMC3899118; doi:10.1371/journal.pone.0085902)
Supplement: Table S2 — Minimum Information About A Microarray Experiment (MIAME) compliance of the experimental design, sampling, hybridization and data analysis. (DOC) [file pone.0085902.s005.doc]

**Table S2**

The following are the details for MIAME compliance:

| **Array Protocol** | |
| --- | --- |
| Protocol Name |  |
| Experiment Description |  |

| **Array Design** | |
| --- | --- |
| Array Name | **Human 40K A+B** |
| No. of Physical Arrays |  |
| Array Version | **1.0** |
| Technology Type  (Selection list) | in situ oligo feature  spotted antibody features  spotted colony features  spotted ds DNA features  spotted protein features  **spotted ss oligo featues**  other  If Other, Specify |
| Substrate Type  (Selection list) | **glass**  nitrocellulose  nylon  silicon  other  If Other, Specify |
| Surface Type  (Selection list) | aminosilane  ploylysine  quartz  other  **epoxy coated glass slide** |
| Attachment Type  (Selection list) | **Covalent**  Electrostatic  other  If Other, Specify |
| Strand Type  (Selection list) | double stranded  **single stranded** |
| Array Protocol |  |
| Date |  |
| Protocol Description |  |

| **Extraction Protocol** | |
| --- | --- |
| Protocol Name |  |
| Extraction  (Selection list) | genomic RNA  mRNA  ployA RNA  **total RNA**  other  If Other, Specify |
| Amplification Method  (Selection list) | PCR  **RNA ploymerase**  other  If Other, Specify |
| Experiment Description |  |

| **Labeling Protocol** | |
| --- | --- |
| Protocol Name | **Dye coupling via monoreactive NHS-ester** |
| Amount of Nucleic Acid Labeled  (Selection list) | **30 ug** |
| Label Used  (Selection list) | Biotin  **CY3**  CY5  32p  33p  other  If Other, Specify |
| Amplification Method  (Selection list) | PCR  **RNA ploymarase**  other  If Other, Specify |
| Experiment Description | **Single channel experiment** |

| **Hybridization Protocol** | |
| --- | --- |
| Protocol Name | **OciChip hybridization protocol** |
| Temperature ^0^C | **50°C** |
| Quantity of Labeled Extract  (Selection list) | **10 μg/slide** |
| Duration  (Selection list) | **2 Hrs**  (in Hours/Minutes/Seconds) |
| Volume  (Selection list) | **100 μl/slide** |
| Hybridization Chamber Type  (Selection list) | Clontech-Atlas Glass  Corning Microarray Technology  Genomic Solutions-GeneTAC solution  genPAK-Genpak and solution  HybStation  Incyte-Microarray Hyb Chamber  in-house built  other  **Tecan HS4800** |
| Experiment Description |  |

| **Scanner Protocol** | |
| --- | --- |
| Protocol Name |  |
| Scanner Type  (Selection list) | Affymetrix 418  **Affymetrix 428**  Affymetrix – GeneChip Scanner 3000  Applied Precision – arrayWoRx  Axon GenePix 4000B  EuroGenTec – ChipReader  GeneFocus – DNAscope Mark II  GeneFocus – Open Frame Research DNA  GeneTAC UC4  Genomic Solution – Genetac 2000  Genomic Solution – LS Iv  in house built  Packard Biochip Technologies – LLC Scanner  Virtek Vision International – Virtek ChipReader  other  If Other, Specify |
| Scanner Software  (Selection list) | **Default Scanner Software**  other  If Other, Specify |
| Experiment Description |  |

| **Image Quantification Protocol** | |
| --- | --- |
| Protocol Name |  |
| Image Quantification Software  (Selection list) | ArrayVision  GenePix  ScanAlyze  Spot  other  **Imagene** |
| Version of Software |  |
| Experiment Description |  |

**Experiment Details**

**Subdivision 1:**

| **Exp Details** | |
| --- | --- |
| Experiment ID | (Created by the System) |
| Experiment Name | (Created by the System) |
| Experiment Type  (Selection list) | **normal vs diseased comparison**  treated vs untreated  dose response  effect of gene knock of  effect of gene knock in  time series  cell cycle  other  If Other, Specify |
| Experiment Variable  (Selection list) | cell type  compound  strain  sex  species  temperature  tissue  **other**  Radiation dose |
| Array Design |  |
| **Contact Details** | |
| Name | Dr Soma Sarkar |
| City | Delhi |
| Email | [soma_sarkar2000@yahoo.com](mailto:soma_sarkar2000@yahoo.com) |
| State | Delhi |
| Phone | +91 11 23883067 |
| Country | India |
| Fax |  |
| Zip |  |
| Experiment Description |  |

**Subdivision 2:**

| **Samples** | |
| --- | --- |
| Sample Name |  |
| Organism Name  (Selection list) | Arabidopsis thaliana  Danio rerio  Drosophila melanogaster  **Homo sapiens**  Mus musculus  Rattus Norvegicus  Saccharomyces cerevisiae  other  If other, Specify |
| BioSource Provider |  |
| Sample Source  (Selection list) | not applicable  **blood**  faeces  frozen section  paraffin section  other  Cell lines |
| Developmental Stage  (Selection list) | **not applicable**  adult  embryo  larva  mixed  pupa  seed  seedling  spore  other  If other, Specify |
| Age  (Selection list) | (days/hours/minutes  /months/seconds/years) |
| Time Point  (Selection list) | birth  hatching  planting  other  If other , Specify |
| Sex  (Selection list) | not applicable  female  hermaphrodite  male  mixed/unknown  other  If other, Specify |
| Genomic Variation  (Selection list) | gene knockout  mutation  transgenic variation  other  If other, Specify |
| Individual Identifier |  |
| Individual Genetic Trait or genotype |  |
| Disease State |  |
| Separation Technique  (Selection list) | embryo sorting  facs  microdissection  trimming  other  If other, Specify |
| Cell Type or Target Cell Type |  |
| Cell Line or Animal Strain or Cultivar |  |
| In Vivo or In Vitro treatment  (Selection list) | not applicable  in vivo treatment  in vitro treatment |
| Organism Part |  |
| Treatment Type  (Selection list) | compound  small molecule  temperature shock  other  If other, Specify |
| Add Clinical Conditions |  |
| **Growth Panel** | |
| Time | (days/hours/minutes  /months/seconds/years) |
| Temperature ^o^C |  |
| Medium |  |

**Subdivision 3**

| **Publications** | |
| --- | --- |
| Publication Status  (Selection list) | in press  not published  online publication  **published** |
| URL |  |
| Journal  (Selection list) | Hum Mol Genet  Immunogenetics  J Bateriol  J Biol Chem  J Cell Biol  J Clin Invest  J Exp Med  J Gen Virol  J Immunol  J Mol Biol  J Mol Evol  J Virol  Mol Biochem Parasitol  Mol Biol Evol  Mol Cell Biol  Mol Gen Genet  Mol Microbiol  Nature  Nucleic Acids Res  Oncogene  Plant Mol Biol  Plant Physiol  Proc Natl Acad Sci U S A  Science  Virology  other  If Others, Specify **PLOS ONE** |
| Volume |  |
| Year |  |
| Title |  |

**Annexure 1**

**Array Design and Printing**

Human 40 K A Ocichip consisting of 20160 spots and Human 40 K B Consisting of 19968 spots were printed on Corning® epoxy coated glass slides using Omnigrid (Gene Machines). Chip was printed using 4x12 SMP4 pins on 75.5x 25.5x1.3mm microarray glass slides. The origin offset values were 0 for X direction and 0 for Y direction. The dot to dot distance is 220 µm; distance between sub-arrays is 150 µm and no of dots per array is 20x21=420. Oligos were printed using spotting buffer A from Ocimum Biosolution at 50% humidity and temperature at 22°C. The time taken by the robotic gridder to print Human 40 K A and B slides altogether was 50 hrs. Each probe was checked for its performance by standard QC process followed at Ocimum Biosolutions. Printed slides were stored in a light-tight box in a bench-top dessicator, with desiccant and were kept at 4°C until they were used for hybridization.

**Annexure 2** (Protocol: Human tissue RNA Extraction)

**Extraction of total RNA and QC check**

RNA concentration was evaluated by measuring absorbance at 260 and 280 nm using a NanoDrop (NanoDrop, USA) and RNA quality was evaluated by electrophoretic analysis with Agilent 2100 Bioanalyzer (Agilent Technologies Inc., Palo Alto, CA). Total RNA that met the quality standards was released for probe generation.

**Annexure 3** (Protocol: Amplification and labelling)

Five microgram of total RNA was used for amplification using Express Art® mRNA amplification Kit Message by linear transcription based RNA amplification system to produce cRNA. Briefly, mRNA is reverse transcribed followed by second strand synthesis priming with a oligo (dT) primer bearing T7 promoter in a thermal cycler using the conditions described in the manual. The resulting cDNA was then purified and was then transcribed with T7 RNA polymerase to generate multiple copies of aminoallyl antisense RNA (aRNA) at 37°C for 16 hrs. aRNA is then labelled with Cy3TM post-labelling reactive dye pack (GE Healthcare, UK) at room temperature and unincorporated Cy3 molecules were removed by purification process.

**Annexure 4** (Hybridization protocol)

20.0 µg of the labeled aRNA in 200 µl of Ocimum's Hyb buffer was denatured at 95°C for 3 mins and 100 μl was used for hybridization with the Human 40 K A and Human 40 K B separately printed at Ocimum Biosolutions, Hyderabad. Hybridization was performed at 50°C for two hrs on Tecan HS 4800 automated Hyb station. Hybridized chips were scanned using Affymetrix 428TMarray scanner at three different PMT gains (40, 50, 60).
